# Neuron-Derived Estrogen Is Critical for Astrocyte Activation and Neuroprotection of the Ischemic Brain

**DOI:** 10.1523/JNEUROSCI.0115-20.2020

**Published:** 2020-09-16

**Authors:** Yujiao Lu, Gangadhara R. Sareddy, Jing Wang, Quanguang Zhang, Fu-Lei Tang, Uday P. Pratap, Rajeshwar R. Tekmal, Ratna K. Vadlamudi, Darrell W. Brann

**Affiliations:** ^1^Department of Neuroscience and Regenerative Medicine, Medical College of Georgia, Augusta University, Augusta, Georgia 30912; ^2^Department of Obstetrics and Gynecology, University of Texas Health, San Antonio, Texas 78229

**Keywords:** 17β-estradiol, aromatase, astrocyte, cerebral ischemia, neuroprotection, stroke

## Abstract

17β-Estradiol (E2) is produced from androgens via the action of the enzyme aromatase. E2 is known to be made in neurons in the brain, but the functions of neuron-derived E2 in the ischemic brain are unclear. Here, we used a forebrain neuron-specific aromatase KO (FBN-ARO-KO) mouse model to deplete neuron-derived E2 in the forebrain and determine its roles after global cerebral ischemia. We demonstrated that ovariectomized female FBN-ARO-KO mice exhibited significantly attenuated astrocyte activation, astrocytic aromatization, and decreased hippocampal E2 levels compared with FLOX mice. Furthermore, FBN-ARO-KO mice had exacerbated neuronal damage and worse cognitive dysfunction after global cerebral ischemia. Similar results were observed in intact male mice. RNA-seq analysis revealed alterations in pathways and genes associated with astrocyte activation, neuroinflammation, and oxidative stress in FBN-ARO-KO mice. The compromised astrocyte activation in FBN-ARO-KO mice was associated with robust downregulation of the astrocyte-derived neurotrophic factors, BDNF and IGF-1, as well as the astrocytic glutamate transporter, GLT-1. Νeuronal FGF2, which acts in a paracrine manner to suppress astrocyte activation, was increased in FBN-ARO-KO neurons. Interestingly, blocking FGF2 signaling by central injection of FGFR3-neutralizing antibody was able to reverse the diminishment in neuroprotective astrocyte reactivity, and attenuate neuronal damage in FBN-ARO-KO mice. Moreover, *in vivo* E2 replacement suppressed FGF2 signaling and rescued the compromised reactive astrogliosis and cognitive deficits. Collectively, our data provide novel genetic evidence for a beneficial role of neuron-derived E2 in astrocyte activation, neuroprotection, and cognitive preservation following ischemic injury to the brain.

**SIGNIFICANCE STATEMENT** Following cerebral ischemia, astrocytes become highly reactive and can exert neuroprotection through the release of neurotrophic factors and clearance of neurotoxic glutamate. The current study advances our understanding of this process by demonstrating that neuron-derived 17β-estradiol (E2) is neuroprotective and critical for induction of reactive astrocytes and their ability to produce astrocyte-derived neurotrophic factors, BDNF and IGF-1, and the glutamate transporter, GLT-1 after ischemic brain damage. These beneficial effects of neuron-derived E2 appear to be due, at least in part, to suppression of neuronal FGF2 signaling, which is a known suppressor of astrocyte activation. These findings suggest that neuron-derived E2 is neuroprotective after ischemic brain injury via a mechanism that involves suppression of neuronal FGF2 signaling, thereby facilitating astrocyte activation.

## Introduction

Traditionally, the steroid hormone, 17β-estradiol (E2) has been considered to be produced primarily in the ovary in females by the enzyme, aromatase. However, a number of studies have shown that aromatase is also expressed in many brain regions, particularly the forebrain in many species, and is capable of producing E2 concentrations that are reportedly even higher than that observed in the peripheral bloodstream (Stoffel-Wagner et al., 1999; Saldanha et al., 2000; Hojo et al., 2004; Ooishi et al., 2012). Under basal conditions, aromatase has been reported to be specifically expressed in neurons in the brain, with little to no expression in glial cells (Yague et al., 2008; Q. G. Zhang et al., 2014). However, in situations of brain injury or ischemia, aromatase has been shown to be robustly induced in reactive astrocytes (Yague et al., 2008; Duncan et al., 2013; Q. G. Zhang et al., 2014; Azcoitia et al., 2018). Two hallmarks have been classically used to define astrocyte reactivity: cellular hypertrophy and overexpression of intermediate filament proteins, such as GFAP, vimentin (Hol and Pekny, 2015), and S100β (Brozzi et al., 2009). Furthermore, transcriptome analysis has implicated two major types of reactive astrocytes: A1 and A2 astrocytes (Zamanian et al., 2012). A1-type astrocytes are induced by inflammatory agents, such as lipopolysaccharide, and have been shown to be proinflammatory and neurotoxic, whereas A2-type astrocytes are induced following cerebral ischemia and have been suggested to be neuroprotective (Liddelow et al., 2017).

With regards to brain-derived E2 functions, previous studies using either global aromatase inhibition or global KO of aromatase have implicated a role of brain-derived E2 in synaptic modulation and neuroprotection (Rune and Frotscher, 2005; Vierk et al., 2012). In addition, central administration of antisense oligonucleotides to knockdown aromatase expression in the brain was shown by our group to be associated with enhanced ischemic injury (Q. G. Zhang et al., 2014). However, these global approaches are difficult to interpret because of their affecting aromatase throughout the body. Furthermore, while the antisense oligonucleotide knockdown approach is more brain-specific, it unfortunately lacks cell specificity, making it difficult to determine the specific role of neuron- versus astrocyte-derived E2 in brain function. Thus, more cell-specific approaches are needed to determine the role of neuron- or astrocyte-derived E2 in brain function. To address this issue, we recently created a forebrain neuron-specific aromatase KO (FBN-ARO-KO) mouse model to help provide novel genetic insight into neuronal E2 actions (Lu et al., 2019). Using the FBN-ARO-KO mice, we previously demonstrated that forebrain neuronal E2 has a critical role in the regulation of forebrain synaptic and dendritic spine density, LTP, and cognitive function in noninjured male and female mice (Lu et al., 2019).

In the current study, we sought to use the FBN-ARO-KO mice to determine the role of neuron-derived E2 in the injured ischemic brain. To accomplish this, we used the mouse two-vessel occlusion global cerebral ischemia (GCI) model. We chose this model because forebrain neurons in the hippocampal CA1 region are highly vulnerable to GCI (Sugawara et al., 2002; Neumann et al., 2013; Q. G. Zhang et al., 2014). In addition, GCI following cardiac arrest and hypotensive shock is associated with significant cognitive deficits, which is thought to be because of damage to the hippocampal CA1 region (Jover et al., 2002; Yang et al., 2010; Neumann et al., 2013). Since our FBN-ARO-KO mouse model has aromatase knocked out in excitatory forebrain neurons, the GCI model thus provides an excellent model for the study of neuron-derived E2 function in the ischemic forebrain.

## Materials and Methods

### 

#### 

##### Animals and GCI

An FBN-ARO-KO mouse model created previously by our group was used in this study (Lu et al., 2019). Three- to 5-month-old age-matched FLOX and FBN-ARO-KO intact male and ovariectomized female mice were subjected to two-vessel occlusion GCI, as previously described (Q. G. Zhang et al., 2008). Bilateral ovariectomy of female mice was conducted 1 week before GCI surgery. For GCI, mice were anesthetized under isoflurane, and both common carotid arteries (CCAs) were isolated and occluded with clips for 20 min. Blood flow through the arteries was verified when the clips were removed. Rectal temperature was maintained at 36.5°C–37.5°C throughout the procedure with a heating blanket. Mice that lost their righting reflex within 30 s, and whose pupils were dilated and unresponsive to light during GCI, were selected for additional experiments. Mice that did not meet these criteria were killed (∼12% of total number). For the animals in sham control, identical procedures were performed, and CCAs were exposed, only without occlusion.

##### Preparation of tissue lysates and Western blotting

Mice were killed under deep anesthesia at 7 d following GCI reperfusion. After completely perfusing with ice-cold 0.9% saline, the hippocampus was quickly collected, and subjected to lysate preparation, as previously described (Lu et al., 2017a). Briefly, hippocampal tissues were homogenized with a motor-driven Teflon homogenizer in 400 µl ice-cold homogenization buffer that contained protease and phosphatase inhibitors, according to the manufacturer's instructions (Thermo Fisher Scientific). The homogenates were then vigorously mixed for 20 min on a rotator at 4°C and centrifuged at 12,000 × *g* for 10 min to get total protein fractions in the supernatant. Total protein concentration was measured with Protein Assay Dye Reagent Concentrate (Bio-Rad). The samples were stored at −80°C until use. Western blotting was conducted as previously described (Han et al., 2015). The primary antibodies used for Western blotting in current study include the following: anti-GFAP (Abcam, catalog #53554, 1:1500), anti-vimentin (Santa Cruz Biotechnology, catalog #sc-7557, 1:200), anti-glutamate transporter-1 (GLT-1, Abcam, catalog #ab106289, 1:1000), anti-FGF2 (Abcam, catalog #ab8880, 1:1000), anti-β-actin (Sigma Millipore, catalog #014M4759, 1:5000), and anti-GAPDH (Santa Cruz Biotechnology, catalog #H2114, 1:2000). Bound proteins were captured using a CCD digital imaging system, and the band intensities were quantified with ImageJ analysis software (version 1.49; National Institutes of Health). Band intensities for the indicated proteins were normalized to the corresponding loading controls to correct the variations in sample loading.

##### Preparation of brain sections and histologic analysis

Mouse brain sections were prepared as described previously (Lu et al., 2017a). Briefly, mice were transcardially perfused with ice-cold 0.9% saline under deep anesthesia at 3, 7, and 14 d after GCI. The mice were killed by decapitation, and the brain was quickly removed and postfixed in 4% PFA at 4°C for 48 h, and cryoprotected in 30% sucrose. The embedded brains in tissue embedding medium (Fisher HealthCare, 4585) were cut into frozen sections of 25 µm thickness in series on a Leica Rm2155 microtome (Leica Microsystems). The following primary antibodies were used for immunofluorescent staining: anti-aromatase (Invitrogen, catalog #PA1-16532, 1:200), anti-GFAP (Abcam, catalog #53554, 1:800), anti-S100β (Sigma Millipore, catalog #AMAB91038, 1:500), anti-BDNF (EMD Millipore, catalog #2865371, 1:200), anti-IGF-1 (Santa Cruz Biotechnology, catalog #SC-9013, 1:50), anti-GLT-1 (Abcam, catalog #ab106289, 1:800), anti-C3D (R&D Systems, catalog #AF2655, 1:200), anti-S100A10 (Invitrogen, catalog #MA5-24769, 1:200), anti-NeuN (EMD Millipore, catalog #mAB377, 1:400), Fluoro-Jade C (F-Jade C, EMD Millipore, catalog #AG325-30MG), anti-MAP2 (Proteintech, catalog #17490-1-AP, 1:800), anti-FGF2 (Abcam, catalog #ab8880, 1:300), and anti-FGFR3 (Invitrogen, catalog #PA5-34574, 1:200). Fluorescent images were captured on an LSM510 Meta confocal laser microscope (Carl Zeiss), using 40× or 63× oil immersion Neofluor objectives with the image size set at 1024 × 1024 pixels. The obtained images were analyzed using LSM510 Meta imaging software, and further subjected to intensity analysis with ImageJ software. IntDen value was measured and expressed as percentage change of their controls. For unbiased comparison across different groups, IntDen measurement was normalized to the cell-free background. Cell counts were conducted manually. All analysis was conducted with unmodified images. At least 4 or 5 randomly selected sections per animal were used for immunostaining, and the typical image was selected for presentation.

##### Construction of astrocyte 3D morphology and measurement of astrocyte volume

To build 3D images of astrocytes in the hippocampal CA1 region, frozen sections of 25 µm thickness were subjected to GFAP staining (Abcam, catalog #53554, 1:800). Confocal single-plane images and *z* stacks with a step size of 0.5 µm were taken under a 63× objective lens of a LSM510 Meta confocal laser microscope (Carl Zeiss). Images were processed with AutoQuant X software, the 3D constructions were obtained, and astrocyte volumes were measured using Imaris software (Bitplane AG). Astrocytes with a cell volume >200 µm^3^ were selected for analysis. At least 4 or 5 randomly selected sections per animal were used for immunostaining, and the typical image was selected for presentation.

##### RNA sequencing analysis

Total RNA was isolated from FLOX and FBN-ARO-KO female ovariectomized mice that underwent 20 min CCA occlusion, followed by 24 h reperfusion. The collected hippocampi samples were subjected to RNA isolation using the RNeasy mini kit (QIAGEN) according to the manufacturer's instructions. Illumina TruSeq RNA sample preparation and sequencing were done using University of Texas Health--San Antonio genomics core facility protocol. Differential gene expression was analyzed by DEseq, and significant genes with adjusted *p* value < 0.05 were used in Ingenuity Pathway Analysis for interpreting biological pathways (Sareddy et al., 2015). The RNA-seq data have been deposited in the GEO database under GEO accession number GSE143836. To further confirm the RNA-seq results, selected genes were examined by real-time qPCR conducted using SYBR Green on an Illumina Real-Time PCR system using the following primers: TDO2-forward: 5′-TGGCAATTACTTGCAGTTGGA-3′, TDO2-reverse: 5′-GTGCTCGTCATGGATTTTGTTC-3′; ST18-forward: 5′-AGCAAAGGGGAACCTGAGTTT-3′, ST18-reverse: 5′-GAACCTCGTTCAGCCTGTAGA-3′; FAM107A-forward: 5′-CAGACCAGAGTACAGAGAGTGG-3′, FAM107A-reverse: 5′-GTGGTTCATAAGCAGCTCACG-3′; HOMER1-forward: 5′-CCCTCTCTCATGCTAGTTCAGC-3′, HOMER1-reverse: 5′-GCACAGCGTTTGCTTGACT-3′; FOS-forward: 5′-CGGGTTTCAACGCCGACTA-3′, FOS-reverse: 5′-TTGGCACTAGAGACGGACAGA-3′; WNT7B-forward: 5′-TTTGGCGTCCTCTACGTGAAG-3′, WNT7B-reverse: 5′-CCCCGATCACAATGATGGCA-3′; S100A10-forward: 5′-TGGAAACCATGATGCTTACGTT-3′, S100A10-reverse: 5′-GAAGCCCACTTTGCCATCTC-3′; PTX-3-forward: 5′-CCTGCGATCCTGCTTTGTG-3′, PTX-3-reverse: 5′-GGTGGGATGAAGTCCATTGTC-3′.

##### Measurement of E2 levels

Hippocampal E2 levels were tested with a high-sensitivity ELISA kit (ADI-900-174, Enzo Life Sciences), as previously described by our group (Q. G. Zhang et al., 2014). Briefly, 100 µl of sample with equal amount of protein was added into each well of the provided microplate, which is coated with a donkey anti-sheep polyclonal antibody; 50 µl of E2 conjugate was subsequently added, accompanied with 50 µl of sheep polyclonal antibody to E2. Afterwards, the plate was covered and incubated for 2 h at room temperature with a shaking speed of ∼500 rpm. The plate was next washed 3 times using the wash buffer, and added with 200 µl of pNpp substrate in each well, followed by another 1 h incubation at room temperature without shaking. Finally, 50 µl of stop solution was added, and the optical density was read at 405 nm on a microplate reader (Bio-Rad). E2 level in each sample was calculated according to the established standard curve and expressed as percentage changes versus FLOX+Sham group.

##### Barnes Maze Test

The Barnes Maze Test was performed to evaluate the hippocampal-dependent spatial reference learning and memory at 7 d after GCI injury, as described in our previous study (Lu et al., 2017b). Briefly, the test was divided into two stages: training trial (days 1-3) and probe trial (day 4). During each day of the training trial, the mice were initially placed in the center of the platform, and trained to find the hidden chamber beneath a target hole for 3 min every day. On the probe trial, the hidden chamber was removed, and replaced with a black board. The escape latency from the center of the maze to the target hole, escape velocity, exploring time in target quadrant, and exploring errors during a 90 s period were recorded using an overhead video camera controlled by ANY-maze video tracking software (Stoelting). The target quadrant is one of the four quadrants in the platform, where the hidden chamber had been located. Exploring error was defined as exploring any hole that did not have the hidden chamber beneath it. The tracking plots and all the parameters were analyzed with ANY-maze software after the experiment.

##### Novel Object Recognition Test

The Novel Object Recognition Test is a well-accepted test for hippocampal-dependent recognition memory in animal models, and was conducted in E2 rescue experiment after the Barnes Maze Test (11 d after GCI) in the current study (Lu et al., 2019). Briefly, this task was divided into two stages: sampling stage (day 1) and choice stage (day 2). One day before the test, the mice were placed in the empty arena for 5 min free exploration for habituation. For the sampling stage, mice were put in the same box, which contains two identical objects with equal distance for 5 min exploring. For the choice stage 24 h later, one of the objects was replaced with a novel object which had identical size, but different shape and appearance with the familiar object. The exploring time of the mice on each object was recorded with ANY-maze video tracking software as mentioned above, and the discrimination index (the percentage of time spent exploring the novel object) was analyzed afterward. Object exploration is defined as the mouse's nose being within 2 cm's range from the object.

##### Open Field Test

The Open Field Test was used to examine locomotor function in mice after GCI following the Barnes Maze Test (11 d after GCI), as described previously (Lu et al., 2019). Mice were put in an open box (56 cm × 56 cm × 45 cm), facing the corner, and allowed to freely explore the apparatus for 5 min. Movement of the mice was recorded with ANY-maze video tracking software as mentioned above. Total travel distance and rearing times during this period were measured.

##### Exogenous E2 rescue experiment

Three-month-old ovariectomized female mice were divided into four groups: FLOX+GCI, KO+GCI, KO+GCI+E2, and KO+GCI+Placebo. For KO+GCI+E2 and KO+GCI+Placebo groups, Alzet osmotic mini-pumps (14 d release; Durect) with E2 (0.0167 mg) or Placebo (20% β-cyclodextrin) were implanted subcutaneously in the upper midback region at the time of ovariectomy as described previously (Lu et al., 2019). The dose of E2 was chosen based on previous results showing that this dose effectively reinstated forebrain E2 levels in FBN-ARO-KO mice back to levels normally observed in the forebrain of FLOX mice (Lu et al., 2019). All mice were subjected to GCI at 7 d after ovariectomy. Barnes Maze and Novel Object Recognition Test behavioral tests were performed at 7 d and 11 d respectively after GCI injury.

##### Intracerebroventricular microinjections of FGFR3 antibody

Previous work has suggested that neuronal FGF2 can inhibit astrocyte activation (Y. Zhang et al., 2017). To determine the role of neuronal FGF2 signaling in mediating the inhibitory action on astrocyte activation in FBN-ARO-KO mice, we administered a FGFR3 blocking antibody (FGFR3 Ab) by intracerebroventricular microinjection as previously reported (Y. Zhang et al., 2017). The following groups were used in our study: (1) FLOX+GCI, (2) KO+GCI, (3) KO+GCI+FGFR3-neutralizing antibody, and (4) KO+GCI+Vehicle. Bilateral intracerebroventricular injection was performed at the time of GCI reperfusion in ovariectomized female FBN-ARO-KO mice, with the following stereotaxic coordinates: 0.6 mm caudal from bregma, 2.00 mm ventral from dura mater, +1.2 mm lateral from the midline. Τhe mice were injected using a dosage of 1 µl of 50 µg/ml of FGFR3 Ab (Invitrogen, PA5-34574) or PBS as vehicle for each lateral ventricle, with a speed of 0.33 µl/min. The syringe was left for 5 min after each injection to allow full diffusion into CSF. The mice were killed 7 d after microinjection, and hippocampal tissues were collected for further histologic analysis.

##### Astrocyte purification

To confirm changes in astrocyte activation and astrocyte phenotypes at 7 d after GCI, astrocytes were isolated from FLOX+Sham, KO+Sham, FLOX+GCI, and KO+GCI ovariectomized female mice brains. To examine astrocyte-derived neurotrophic/neuroprotective factors in FBN-ARO-KO+GCI mice following FGFR3 neutralization, astrocytes were further isolated from FLOX+GCI, KO+GCI, KO+GCI+Vehicle, and KO+GCI+FGFR3 Ab mice at 7 d after FGFR3 antibody intracerebroventricular injection. First, an Adult Brain Dissociation Kit (Miltenyi Biotec, catalog #130-107-677) was used to dissociate brain tissue into single-cell suspensions according to the manufacturer's instructions. Briefly, mice were killed under anesthesia with the brain quickly removed and stored in cold D-PBS. Brain tissues were cut into 0.5 cm pieces using a scalpel, and then transferred into gentleMACS C tubes, which contained 1950 µl of enzyme mix 1 solution; 30 µl of enzyme mix 2 was added into C tubes afterward. The tightly closed C tubes were then attached onto a gentleMACS Octo Dissociator with Heater for tissue dissociation. The obtained samples were filtered with a MACS SmartStrainer (70 µm) and subjected to debris removal and red blood cell removal.

The single-cell suspension obtained from above steps was subjected to magnetic labeling with an Anti-ACSA-2 MicroBead Kit (Miltenyi Biotec, catalog #130-097-678) according to the manufacturer's instructions. Briefly, the cell suspension was blocked with 10 µl of FcR blocking reagent per 10^7^ total cells at 4°C for 10 min; 10 µl of Anti-ACSA-2 MicroBeads per 10^7^ total cells was added and incubated at 4°C for 15 min. The cells were washed by adding 2 ml of PB buffer per 10^7^ total cells and resuspended in 500 µl PB buffer per 10^7^ total cells. The cell suspension was applied to an MS column (Miltenyi Biotec, catalog #130-042-201) to get purified astrocytes. The purity of astrocytes obtained by this procedure was determined by flow cytometry. The cell population was presented in a dot plot with four quadrants. The percentage of ACSA-2-positive cells was defined as astrocyte purity, which was 94%, and is consistent with previous reports (Feldmann et al., 2014).

Protein lysates of the purified astrocytes were made for further Western blotting analysis. Primary antibodies used here include the following: anti-GFAP (Abcam, catalog #53554, 1:1500), anti-vimentin (Santa Cruz Biotechnology, catalog #sc-7557, 1:200), anti-S100β (Sigma Millipore, catalog #AMAB91038, 1:1000), anti-C3D (R&D Systems, catalog #AF2655, 1:1000), anti-FKBP5 (Thermo Fisher Scientific, catalog #PA1-020, 1:1000), anti-GBP2 (Abcam, catalog #ab203238, 1:1000), anti-S100A10 (Invitrogen, catalog #MA5-24769, 1:1000), anti-PTX3 (Abcam, catalog #ab125007, 1:1000), anti-TGM1 (Abcam, catalog #ab103814, 1:1000), anti-GLT-1 (Abcam, catalog #ab106289, 1:1000), anti-Aromatase (Invitrogen, catalog #PA1-16532, 1:1000), anti-p-STAT3 (Cell Signaling Technology, catalog #9131S, 1:1000), anti-STAT3 (Cell Signaling Technology, catalog #9139S, 1:1000), anti-BDNF (EMD Millipore, catalog #2865371, 1:1000), anti-β-actin (Sigma Millipore, catalog #014M4759, 1:5000), and anti-GAPDH (Santa Cruz Biotechnology, catalog #H2114, 1:2000).

##### Experimental design and statistical analyses

All experiments in the current study were performed on age-matched FLOX control and FBN-ARO-KO mice. Both intact males and ovariectomized females were used, and sham animals were used as controls. We used independent groups of animals to conduct the biochemical/histologic experiments and behavioral tests. Eight to 11 animals per group were used for behavioral tests; otherwise, 3–6 independent samples from each group were analyzed. Investigators were blinded to the groups for the behavioral assessments. A Student's *t* test was conducted when only comparing two groups. In experiments with two interaction factors and requiring multiple group comparisons, two-way ANOVA was performed followed by Tukey's all pairwise comparisons test to determine group difference. All the data were analyzed with SigmaStat 3.5 software. Data represented in bar graphs were expressed as mean ± SE. *p* < 0.05 was considered statistically significant.

## Results

### Loss of forebrain neuron-derived E2 leads to attenuated astrocyte activation and aromatization

**Figure 1.**
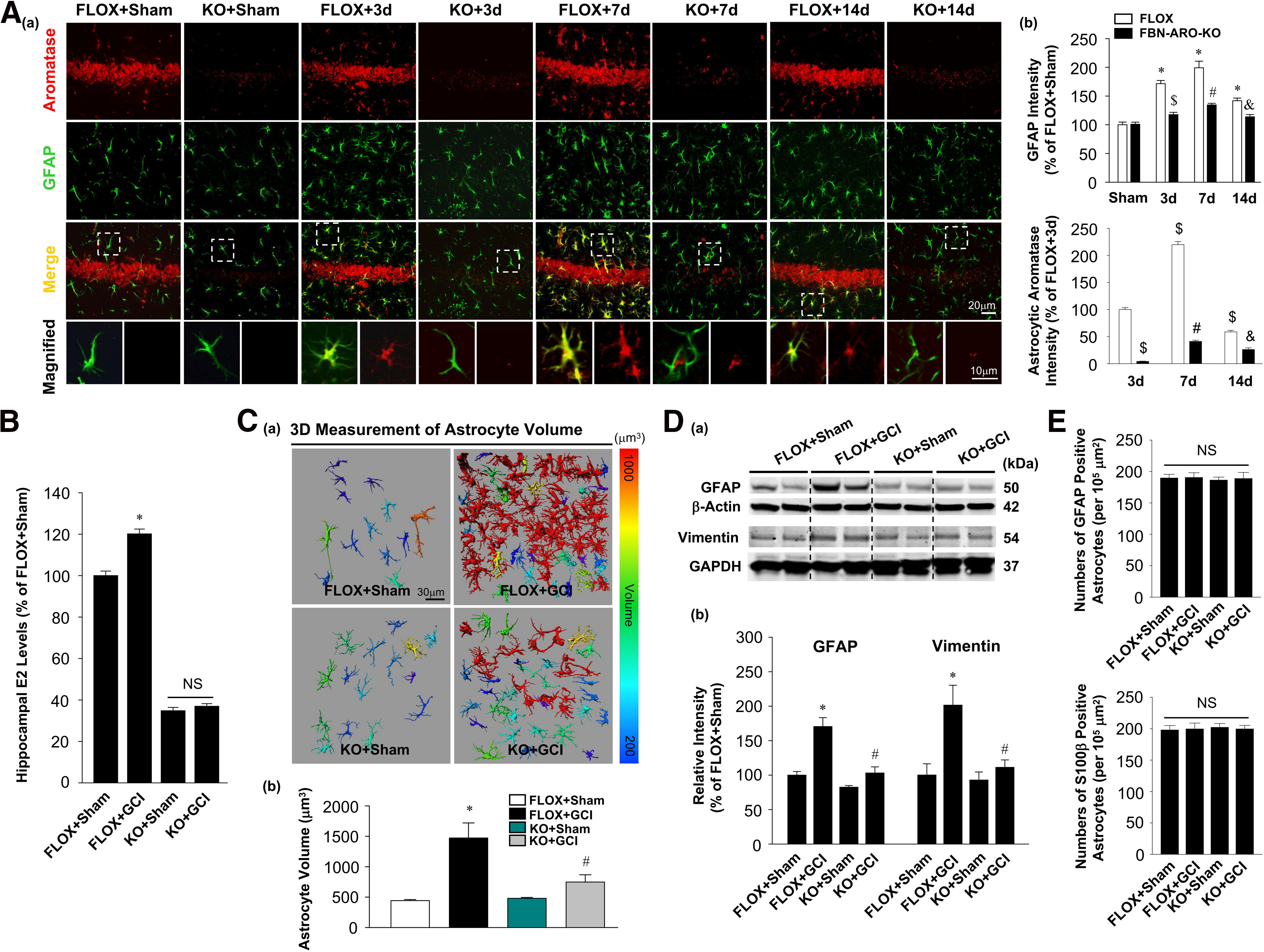
Attenuated astrocyte activation and aromatization in ovariectomized female FBN-ARO-KO mice after GCI. ***A***, IHC analysis for alterations in astrocyte reactivity and aromatase induction in hippocampal CA1 region at 3, 7, and 14 d after GCI. ***B***, Measurement of hippocampal E2 levels with high-sensitivity E2 ELISA kit at 7 d after GCI. ***Ca***, Representative 3D images of hippocampal CA1 astrocytes with cell body volumes distinguished with different colors. ***Cb***, Astrocyte volumes from the indicated groups were measured and quantitatively analyzed. ***D***, Astrocyte activation was confirmed by examining the expression of two typical astrocyte markers, GFAP and vimentin, with Western blot analysis. ***E***, Astrocyte numbers in hippocampal CA1 region were counted with both GFAP and S100β immunostaining and expressed as cell numbers per 10^5^ µm^2^. Values are mean ± SEM of determinations from each group. *N* = 3-5. *p < 0.05 versus FLOX+Sham. ^$^*p* < 0.05 versus FLOX + 3 d. ^#^*p* < 0.05 versus FLOX + 7 d. ^&^*p* < 0.05 versus FLOX + 14 d. NS, no significant difference.

It is known that aromatase can be induced in reactive astrocytes of injured brains (Garcia-Segura et al., 1999; Q. G. Zhang et al., 2014). Therefore, we first examined astrocyte activation and astrocytic aromatase expression in the hippocampal CA1 region of ovariectomized female FLOX and FBN-ARO-KO mice at different time points (3, 7, and 14 d) after GCI by performing GFAP and aromatase double staining (Fig. 1*Aa*). Quantification of GFAP intensity in the hippocampal CA1 region showed that GFAP levels were strongly increased in FLOX mice after GCI (*F* = 82.404, *p* < 0.001 for FBN-ARO-KO; *F* = 48.592, *p* < 0.001 for GCI; *F* = 13.292, *p* < 0.001 for FBN-ARO-KO and GCI interaction; *p* < 0.001 for 3 d, *p* < 0.001 for 7 d, *p* < 0.001 for 14 d; *n* = 4, two-way ANOVA), suggesting robust activation and reactivity of hippocampal astrocytes after ischemia (Fig. 1*Ab*). Interestingly, we observed the highest GFAP level at 7 d after GCI compared with 3 and 14 d, suggesting that the strongest astrocyte activation occurs at this time point in our model (Fig. 1*Ab*). Importantly, we found that GFAP intensity in FBN-ARO-KO+GCI mice was robustly decreased compared with FLOX+GCI group at all three time points (*p* < 0.001 for 3, 7, and 14 d; *n* = 4, two-way ANOVA). This finding indicates that loss of neuron-derived E2 causes astrocytes to be less activated after GCI compared with astrocytes from FLOX+GCI mice. GFAP and aromatase double staining further showed that aromatase was specifically localized in hippocampal CA1 neurons in FLOX+Sham mice, and that aromatase expression is strongly decreased in FBN-ARO-KO+Sham mice compared with FLOX+Sham mice. Aromatase was not detected in the resting astrocytes from either group. However, it was robustly induced in astrocytes of FLOX+GCI mice (*F* = 1390.119, *p* < 0.001 for FBN-ARO-KO; *F* = 413.034, *p* < 0.001 for GCI; *F* = 237.575, *p* < 0.001 for FBN-ARO-KO and GCI interaction; *p* < 0.001 for 7 d vs 3 d, *p* = 0.026 for 14 d vs 3 d; *n* = 4, two-way ANOVA). In contrast, aromatase levels in astrocytes from FBN-ARO-KO+GCI mice were significantly decreased compared with their FLOX+GCI controls (Fig. 1*Ab*; *p* < 0.001 for 3, 7, and 14 d; *n* = 4, two-way ANOVA).

We next measured hippocampal E2 levels in ovariectomized female FLOX and FBN-ARO-KO mice 7 d after GCI using a high-sensitivity ELISA kit. The results revealed that, in FLOX mice, there was a significant increase in hippocampal E2 levels at 7 d after GCI compared with FLOX+Sham controls (Fig. 1*B*; *F* = 1542.173, *p* < 0.001 for FBN-ARO-KO; *F* = 34.889, *p* < 0.001 for GCI; *F* = 22.669, *p* < 0.001 for FBN-ARO-KO and GCI interaction; *p* < 0.001, *n* = 4, two-way ANOVA). Examination of FBN-ARO-KO mice revealed that FBN-ARO-KO+Sham mice displayed a ∼66% loss of hippocampal E2 levels compared with FLOX+Sham controls (*p* < 0.001, *n* = 4, two-way ANOVA), a finding in agreement with our previous study (Lu et al., 2019). Furthermore, in contrast to FLOX+GCI mice, FBN-ARO-KO+GCI mice failed to show any significant increase in hippocampal E2 production after GCI (Fig. 1*B*; *p* = 0.328, *n* = 4, two-way ANOVA**)**.

To further analyze and quantitate the alterations of astrocyte morphology/reactivity, we constructed 3D images of astrocytes in the hippocampal CA1 region and measured the cell body volumes of the astrocytes using Imaris software (Ardalan et al., 2017) (Fig. 1*C*). The results revealed that there was no difference in astrocyte volume between FLOX+Sham and FBN-ARO-KO+Sham mice (*F* = 6.182, *p* = 0.024 for FBN-ARO-KO; *F* = 22.102, *p* < 0.001 for GCI; *F* = 7.591, *p* = 0.014 for FBN-ARO-KO and GCI interaction; *p* = 0.136, *n* = 5, two-way ANOVA), indicating a resting state in both groups. However, astrocyte volumes of FLOX+GCI mice were strongly increased compared with FLOX+Sham controls (*p* = 0.008, *n* = 5, two-way ANOVA), indicating a robust induction of reactive astrocytes. In contrast, astrocytes volumes in FBN-ARO-KO+GCI mice were not significantly elevated (*p* = 0.095, *n* = 5, two-way ANOVA) comparing with their sham control, and were significantly lower than FLOX+GCI mice (*p* = 0.03, *n* = 5, two-way ANOVA). This finding suggests that induction of reactive astrocytes after global brain ischemia is significantly compromised in FBN-ARO-KO mice. To further confirm the results, we conducted Western blot analysis to examine the expression of two typical astrocyte markers, GFAP and vimentin (Fig. 1*D*). Both GFAP (∼70% increase, *F* = 19.358, *p* = 0.001 for FBN-ARO-KO; *F* = 22.181, *p* < 0.001 for GCI; *F* = 6.643, *p* = 0.028 for FBN-ARO-KO and GCI interaction; *p* = 0.007, *n* = 4, two-way ANOVA) and vimentin (∼101% increase, *F* = 6.963, *p* = 0.03 for FBN-ARO-KO; *F* = 10.542, *p* = 0.012 for GCI; *F* = 5.065, *p* = 0.045 for FBN-ARO-KO and GCI interaction; *p* = 0.038, *n* = 3, two-way ANOVA) levels were robustly elevated in the hippocampus of FLOX+GCI versus FLOX+Sham controls at 7 d after GCI. In contrast, FBN-ARO-KO+GCI mice did not display significant increase (20% for GFAP, *p* = 0.115, *n* = 4; 18% for vimentin, *p* = 0.312, *n* = 3, two-way ANOVA) versus FBN-ARO-KO+Sham controls, indicating strongly diminished reactive astrogliosis in the FBN-ARO-KO mice after GCI. To determine whether a change in astrocyte number might contribute to these alterations, we counted astrocyte numbers in the hippocampal CA1 region using immunostaining for GFAP, as well as for S100β, another typical marker of astrocytes (Fig. 1*E*). Analysis for GFAP- and S100β-positive cells showed no differences among the indicated groups, suggesting that only astrocyte activation, not astrocyte loss or proliferation, was diminished after neuronal E2 depletion in our GCI model.

### FBN-ARO-KO mice exhibit enhanced neuronal damage and greater cognitive deficits after GCI

**Figure 2.**
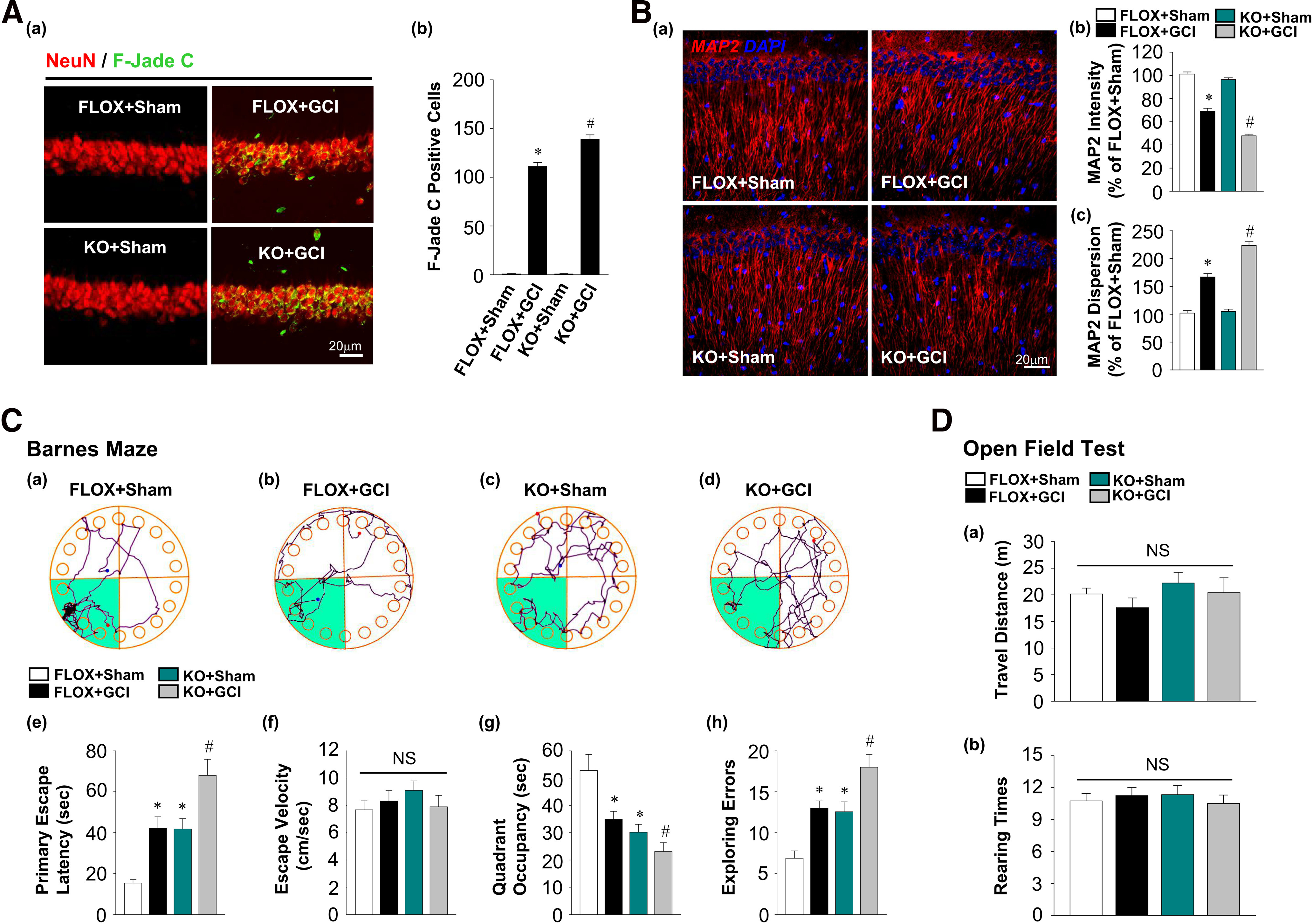
Ovariectomized female FBN-ARO-KO mice exhibit worse neuronal damage and cognitive deficits 7 d after GCI injury. ***Aa***, IHC analysis for degenerating neurons by F-Jade C, a typical neurodegenerative marker, with NeuN double staining. ***Ab***, The number of F-Jade C-positive neurons per 250 um was counted. ***B***, MAP2 staining was performed to further evaluate hippocampal CA1 dendrite morphology following aggravated neuronal damage in FBN-ARO-KO mice. ***C***, Barnes Maze Test was used to assess the spatial reference memory. Representative track plots of FLOX+Sham (***Ca***), FLOX+GCI (***Cb***), KO+Sham (***Cc***), and KO+GCI (***Cd***) on the probe trial were recorded. Primary escape latency (***Ce***), escape velocity (***Cf***), quadrant occupancy (***Cg***), and exploring errors (***Ch***) during probe trial were analyzed. ***D***, Open field test was conducted to determine the locomotor ability, in which the travel distance and rearing times were recorded and analyzed. Values are mean ± SEM of determinations from each group. *N* = 5 for ***A***, ***B***; *N* = 8 or 9 for ***C***, ***D***. *p < 0.05 versus FLOX+Sham. ^#^*p* < 0.05 versus FLOX+GCI. NS, no significant difference.

Next, we investigated the neuronal damage and functional outcome of ovariectomized female FBN-ARO-KO mice at 7 d after GCI reperfusion. First, double staining in the hippocampal CA1 region for NeuN, a neuronal marker, and F-Jade C, a marker of neuronal degeneration, was performed (Schmued et al., 2005), and the number of F-Jade C-positive neurons per 250 µm of the hippocampal CA1 region was counted. It revealed that FBN-ARO-KO+GCI mice had significantly increased F-Jade C-positive hippocampal pyramidal neurons compared with FLOX+GCI mice, suggesting enhanced neuronal degeneration (Fig. 2*A*; *F* = 20.534, *p* < 0.001 for FBN-ARO-KO; *F* = 1616.096, *p* < 0.001 for GCI; *F* = 20.534, *p* < 0.001 for FBN-ARO-KO and GCI interaction; *p* = 0.002, *n* = 5, two-way ANOVA). No apparent F-Jade C staining was observed in FLOX and FBN-ARO-KO sham mice. Further examination of alterations in neuronal structure by MAP2 staining showed that FBN-ARO-KO+GCI mice had less MAP2 intensity (*F* = 42.487, *p* < 0.001 for FBN-ARO-KO; *F* = 422.714, *p* < 0.001 for GCI; *F* = 17.086, *p* < 0.001 for FBN-ARO-KO and GCI interaction; *p* < 0.001, *n* = 5, two-way ANOVA) and greater dispersion (*F* = 29.739, *p* < 0.001 for FBN-ARO-KO; *F* = 283.714, *p* < 0.001 for GCI; *F* = 24.332, *p* < 0.001 for FBN-ARO-KO and GCI interaction; *p* < 0.001, *n* = 5, two-way ANOVA) than FLOX+GCI mice (Fig. 2*B*), suggesting poorer neuronal structural integrity in FBN-ARO-KO mice compared with FLOX mice after GCI.

We next examined functional outcome in FBN-ARO-KO+GCI mice by performing behavioral tests. First, the Barnes Maze Test was conducted to assess hippocampal-dependent spatial reference memory (Fig. 2*C*). Results in the probe trial showed that FLOX+GCI mice displayed a significant increase in escape latency (*F* = 22.64, *p* < 0.001 for FBN-ARO-KO; *F* = 23.468, *p* < 0.001 for GCI; *F* = 3.55, *p* = 0.035 for FBN-ARO-KO and GCI interaction; *p* = 0.002, *n* = 8, two-way ANOVA) and exploring errors (*F* = 20.679, *p* < 0.001 for FBN-ARO-KO; *F* = 24.264, *p* < 0.001 for GCI; *F* = 8.4, *p* = 0.027 for FBN-ARO-KO and GCI interaction; *p* < 0.001, *n* = 8, two-way ANOVA), and a robust decrease in quadrant occupancy (*F* = 19.562, *p* < 0.001 for FBN-ARO-KO; *F* = 10.247, *p* = 0.003 for GCI; *F* = 9.136, *p* = 0.018 for FBN-ARO-KO and GCI interaction; *p* = 0.017, *n* = 8, two-way ANOVA) compared with FLOX+Sham mice, indicative of impaired spatial reference memory retention. FBN-ARO-KO+Sham mice also displayed cognitive deficits compared with FLOX+Sham mice, a finding consistent with our previous study (Lu et al., 2019). Interestingly, FBN-ARO-KO+GCI mice exhibited an even greater increase in escape latency, exploring errors, and decrease in quadrant occupancy compared with FLOX+GCI mice (*p* = 0.018, *p* = 0.014, and *p* = 0.017 respectively, *n* = 8, two-way ANOVA). Identical escape velocities among all groups demonstrated that the altered parameters discussed above were not because of speed variations. We also used the Open Field Test to evaluate locomotor function by recording the total travel distance and rearing times in the open arena (Fig. 2*D*). However, no differences in these two parameters were observed among the indicated groups, suggesting that loss of forebrain neuronal E2 does not affect locomotor ability under normal and GCI conditions.

### RNA-Seq analysis reveals unique gene profiles and pathways regulated by neuron-derived E2 after GCI

**Figure 3.**
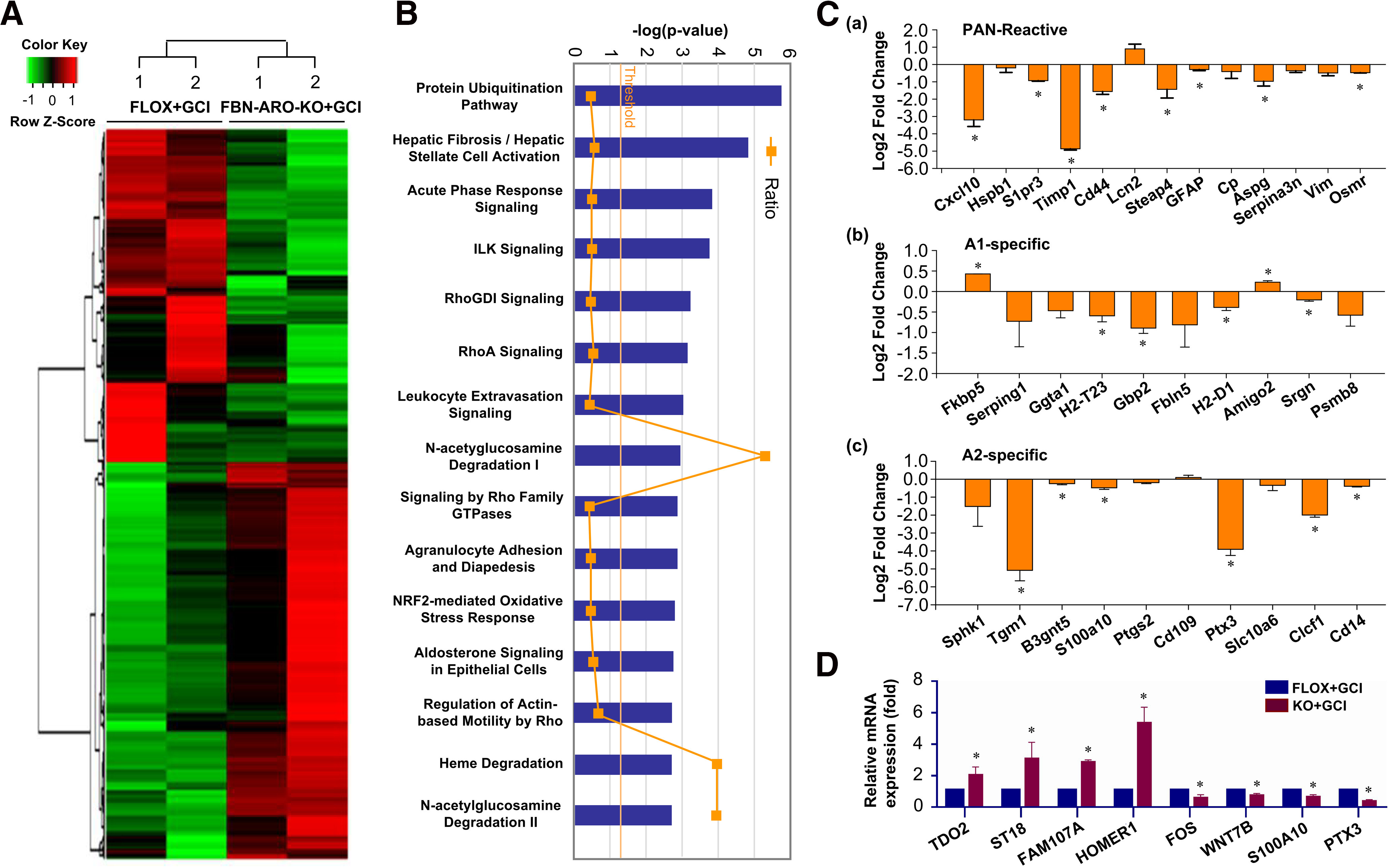
RNA-sequencing analysis of hippocampal transcriptome alterations in ovariectomized female FBN-ARO-KO mice after GCI. ***A***, Representative heat map of differentially expressed genes from FLOX+GCI and FBN-ARO-KO+GCI groups at 24 h after GCI insult. ***B***, Differentially expressed genes with adjusted *p* value < 0.05 were examined by Ingenuity Pathway Analysis, and the top canonical pathways are shown. ***C***, Top astrocyte pan-reactive (***Ca***), A1-specific (***Cb***), and A2-specific (***Cc***) transcripts that were altered in FBN-ARO-KO mice. ***D***, Selected differentially expressed genes that are associated with neuroprotective astrocyte activation, neuroinflammation, and apoptosis were confirmed by qRT-PCR. Values are mean ± SEM of determinations from each group. *p < 0.05 versus FLOX+GCI.

To identify gene profiles and pathways regulated by forebrain neuronal E2 after GCI injury, we performed global transcriptome analysis. Twenty-four hours after GCI reperfusion, RNA samples were isolated from the hippocampus of ovariectomized female FLOX+GCI and FBN-ARO-KO+GCI mice and subjected to RNA-Seq analysis as described previously (Sareddy et al., 2015). A representative heat map depicting gene changes across the groups is shown in Figure 3*A*. Significant genes with adjusted *p* value < 0.05 were selected for analysis. Differentially expressed genes were subjected to pathway analysis using Ingenuity Pathway Analysis software, and the top canonical pathways identified are shown in Figure 3*B*. We observed that some of the key pathways that regulate astrocyte reactivity, such as RhoA signaling, actin-based motility by Rho signaling, signaling by Rho family GTPases, and NRF2-mediated oxidative stress response, were significantly altered in FBN-ARO-KO+GCI mice. However, it should be noted that RhoA signaling also plays roles in nonastrocyte cells, particularly neurons, and E2 can also regulate Rho signaling in neurons (Kramar et al., 2009).

To determine whether there were changes in transcription of genes involved in astrocyte reactivity, we screened astrocyte-associated gene profiles for the pan-reactive, proinflammatory A1-type and neuroprotective A2-type astrocyte phenotypes. The results revealed a significant downregulation of top pan-reactive astrocyte transcripts in FBN-ARO-KO+GCI mice (Fig. 3*Ca*), as well as strong downregulation of most of the astrocyte A2-specific transcripts in FBN-ARO-KO+GCI mice (Fig. 3*Cc*). Some of the astrocyte A1-specific transcripts also showed significant alterations in FBN-ARO-KO+GCI mice, with several showing a significant decrease, while a few showed a significant increase (Fig. 3*Cb*). Finally, we used qRT-PCR to validate key genes implicated to regulate astrocyte activation, neuroinflammation, and apoptosis (Fig. 3*D*). The results confirmed significant upregulation of genes implicated in neuroinflammation (*t* = −2.268, *p* = 0.047, *n* = 6 for TDO2; *t* = −17.714, *p* < 0.001, *n* = 6 for Fam107a; *t* = −4.552, *p* = 0.001, *n* = 6 for Homer1; unpaired *t* test) and apoptosis (*t* = −2.138, *p* = 0.038, *n* = 6 for ST18, unpaired *t* test), and robust downregulation of genes regulating astrocyte “A2” phenotype (*t* = 3.281, *p* = 0.046, *n* = 3 for Fos; *t* = 40, *p* = 0.008, *n* = 5 for S100A10; *t* = 57, *p* = 0.002, *n* = 6 for PTX3; unpaired *t* test) and synapse maturation (*t* = 3.948, *p* = 0.029, *n* = 3 for WNT7B; unpaired *t* test). The results suggest that neuronal E2 regulates transcription of genes and pathways involved in astrocyte activation and neuroprotection after global brain ischemia.

### Loss of forebrain neuronal E2 leads to reduced astrocyte A2 phenotype

**Figure 4.**
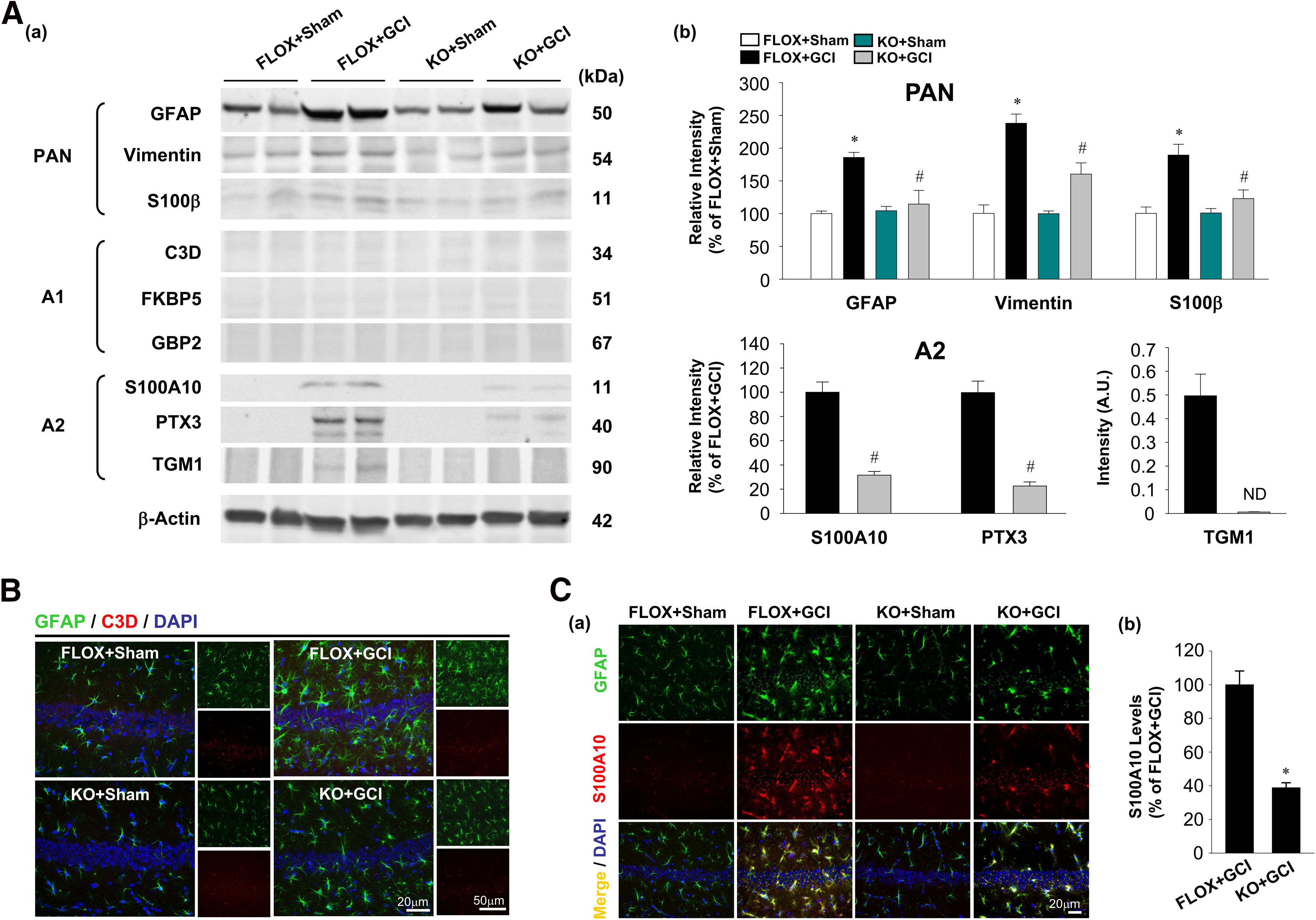
Ovariectomized female FBN-ARO-KO mice have diminished astrocyte A2 phenotype 7 d after GCI. ***Aa***, Astrocyte PAN-reactive, A1-specific, and A2-specific phenotypes were determined by Western blot analysis with purified astrocyte lysates from ovariectomized female brains 7 d after GCI reperfusion. ***Ab***, Levels of the examined markers for different astrocyte phenotypes were quantitatively analyzed. *N* = 3. ***B***, IHC analysis for astrocyte A1 phenotype by double staining of the selected astrocyte A1 marker C3D with GFAP. ***Ca***, IHC examination for astrocyte A2 phenotype using A2-specific marker S100A10 and GFAP double staining. ***Cb***, Percentage changes of S100A10 in FBN-ARO-KO+GCI mice versus FLOX+GCI mice were quantified. *N* = 4. Values are mean ± SEM of determinations from each group. *p < 0.05 versus FLOX+Sham. ^#^*p* < 0.05 versus FLOX+GCI. ND, Nondetectable.

To further validate the astrocyte phenotypes (PAN, A1, and A2) at 7 d after GCI, we isolated astrocytes from the ovariectomized female brains with an Anti-ACSA-2 MicroBead Kit and collected protein lysates from the purified astrocytes for Western blot analysis. First, PAN-specific astrocyte phenotype was determined using three typical markers: GFAP, vimentin, and S100β (Fig. 4*Aa*). The result displayed a consistent pattern with our previous data using whole hippocampus analysis. As shown in Figure 4*Ab*, FLOX+GCI astrocytes have significantly increased GFAP (*F* = 8.067, *p* = 0.022 for FBN-ARO-KO; *F* = 16.642, *p* = 0.004 for GCI; *F* = 10.332, *p* = 0.012 for FBN-ARO-KO and GCI interaction; *p* < 0.001, *n* = 3, two-way ANOVA), vimentin (*F* = 9.019, *p* = 0.017 for FBN-ARO-KO; *F* = 58.118, *p* < 0.001 for GCI; *F* = 8.866, *p* = 0.018 for FBN-ARO-KO and GCI interaction; *p* < 0.001, *n* = 3, two-way ANOVA), and S100β (*F* = 7.331, *p* = 0.027 for FBN-ARO-KO; *F* = 20.946, *p* = 0.002 for GCI; *F* = 7.631, *p* = 0.025 for FBN-ARO-KO and GCI interaction; *p* = 0.01, *n* = 3, two-way ANOVA) compared with FLOX+Sham control astrocytes, suggesting a robust astrocyte activation following brain ischemia. In contrast, FBN-ARO-KO+GCI astrocytes had a pronounced decrease in all of these markers compared with FLOX+GCI astrocytes (*p* = 0.033 for GFAP, *p* = 0.025 for vimentin, *p* = 0.035 for S100β, *n* = 3, two-way ANOVA), indicating attenuated astrocyte reactivity after loss of neuronal-derived E2.

Next, induction of the astrocyte A1 phenotype was examined by Western blot analysis using antibodies to three A1-selective markers, C3D, FKBP5, and GBP2. However, as shown in Figure 4*Aa*, we did not detect expression of these A1-selective marker proteins in any of the groups, suggesting that the A1 phenotype is not induced in FLOX+GCI or FBN-ARO-KO+GCI astrocytes in our ischemia model (Fig. 4*Aa*). We next examined induction of the astrocyte A2 phenotype using antibodies specific to S100A10, PTX3, and TGM1: A2-selective genes that showed significant changes in our RNA-sequencing analysis results. Interestingly, all three markers, which were not expressed in FLOX and FBN-ARO-KO Sham astrocytes, had strong induction in FLOX+GCI astrocytes (Fig. 4*Aa*,*Ab*). In contrast, astrocytes from FBN-ARO-KO+GCI mice had robust downregulation of the A2-selective marker proteins S100A10 (*t* = 7.618, *p* = 0.002, *n* = 3, unpaired *t* test) and PTX3 (*t* = 7.702, *p* = 0.002, *n* = 3, unpaired *t* test). Furthermore, while TGM1 expression was robustly expressed in the FLOX+GCI group, its expression was not detectible in the FBN-ARO-KO+GCI group (Fig. 4*Aa*,*Ab*). These results indicate that FBN-ARO-KO+GCI mice have strongly reduced A2 astrocyte polarization compared with FLOX+GCI mice.

Finally, we next sought to confirm the above results with IHC analysis. Double immunostaining was performed in ovariectomized female mice at 7 d after GCI in the hippocampal CA1 region for GFAP and C3D, the selected marker of A1 astrocyte. C3D expression was not detected in any of the indicated groups (Fig. 4*B*), consistent with the lack of expression observed in purified astrocytes described above. In contrast, S100A10, the marker of A2 astrocyte, was demonstrated to be strongly induced and colocalized with GFAP in hippocampal astrocytes of FLOX+GCI mice at 7 d after GCI. Examination of FBN-ARO-KO+GCI mice revealed a significant reduction of S100A10 level in the hippocampal CA1 region compared with FLOX+GCI mice (Fig. 4*C*; *t* = 7.077, *p* < 0.001, *n* = 4, unpaired *t* test), suggesting a compromised A2 astrocyte induction after GCI in mice deficient in forebrain neuronal E2.

### Depletion of forebrain neuronal E2 invokes compromised astrocyte functions

**Figure 5.**
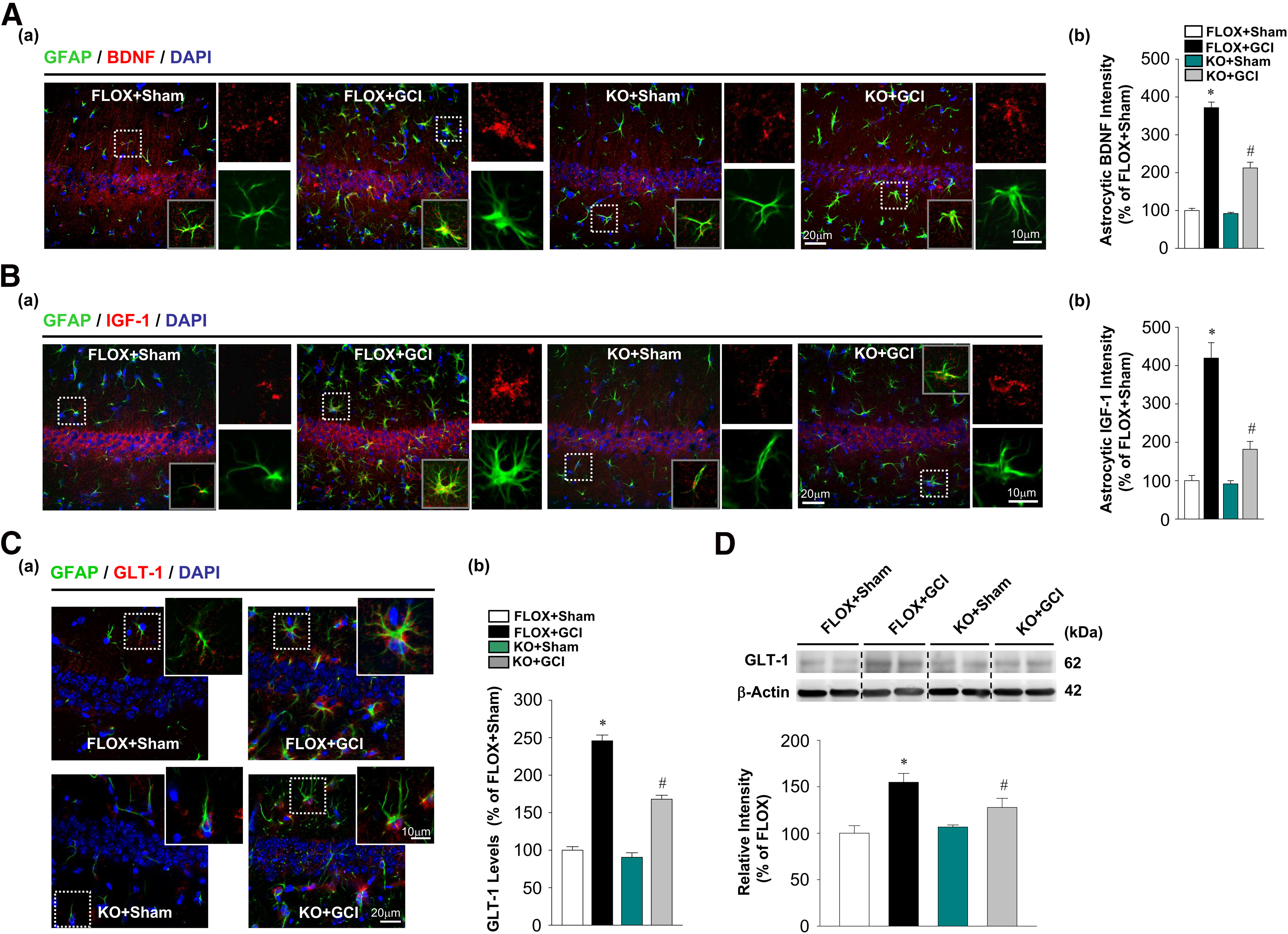
Loss of neuronal E2 attenuates the expression of astrocytic neurotrophins and glutamate transporter in ovariectomized female FBN-ARO-KO mice 7 d after GCI. ***Aa***, Levels of astrocyte-derived BDNF were determined by GFAP and BDNF costaining. ***Ab***, BDNF intensity in astrocytes was further quantitatively analyzed. ***Ba***, Immunofluorescent image of IGF-1 and GFAP demonstrated a pronounced reduction of IGF-1 expression in astrocytes in FBN-ARO-KO+GCI mice when comparing with FLOX+GCI control. ***Bb***, IGF-1 intensity in astrocytes was further quantitatively analyzed. ***Ca***, Levels of glutamate transporter in astrocytes following compromised astrocyte reactivity in FBN-ARO-KO+GCI mice were interrogated by GLT-1 and GFAP costaining. ***Cb***, GLT-1 production was quantified. ***D***, GLT-1 alterations were further validated by Western blot analysis. Values are mean ± SEM of determinations from each group. *N* = 4 or 5. *p < 0.05 versus FLOX+Sham. ^#^*p* < 0.05 versus FLOX+GCI.

Reactive astrocyte induction after cerebral ischemia has been implicated to be neuroprotective, due in part to enhanced production of neurotrophic factors, as well as astrocyte glutamate transporters that clear excess neurotoxic glutamate generated after cerebral ischemia (Z. Liu and Chopp, 2016). Therefore, we next examined expression of the astrocytic neurotrophins BDNF and IGF-1 in the hippocampal CA1 region using double IHC analysis with antibodies for either BDNF or IGF-1 and GFAP. Three astrocytes in each hippocampal CA1 region were randomly selected for analysis. Astrocytic BDNF and IGF-1 intensities were quantitatively analyzed and expressed as percent change of each group versus FLOX+Sham. The results in Figure 5*A* revealed that BDNF expression in hippocampal astrocytes was strongly increased in FLOX+GCI mice at 7 d after GCI compared with FLOX+Sham (*F* = 58.198, *p* < 0.001 for FBN-ARO-KO; *F* = 319.94, *p* < 0.001 for GCI; *F* = 47.658, *p* < 0.001 for FBN-ARO-KO and GCI interaction; *p* < 0.001, *n* = 4, two-way ANOVA). In contrast, in the less activated astrocytes in FBN-ARO-KO+GCI mice, astrocytic BDNF levels were significantly diminished compared with FLOX+GCI mice (*p* < 0.001, *n* = 4, two-way ANOVA). No difference in astrocyte-localized BDNF was found in the resting astrocytes between FLOX+Sham and FBN-ARO-KO+Sham (*p* = 0.265, *n* = 4, two-way ANOVA). Similarly, astrocytic IGF-1 expression in the hippocampal CA1 region of FBN-ARO-KO+GCI mice at 7 d after GCI was significantly attenuated compared with FLOX+GCI mice (Fig. 5*B*; *F* = 26.398, *p* < 0.001 for FBN-ARO-KO; *F* = 73.146, *p* < 0.001 for GCI; *F* = 22.93, *p* < 0.001 for FBN-ARO-KO and GCI interaction; *p* < 0.001, *n* = 4, two-way ANOVA). There was no difference in astrocytic IGF-1 expression between FLOX sham versus FBN-ARO-KO sham mice (*p* = 0.809, *n* = 4, two-way ANOVA). Finally, we examined for differences in astrocyte expression of the astrocytic GLT-1 at 7 d following GCI injury. Double IHC analysis for GLT-1 and GFAP revealed that GLT-1 levels were robustly upregulated in FLOX+GCI-reactive astrocytes in the hippocampal CA1 region compared with the resting astrocytes in FLOX+Sham mice (Fig. 5*C*; *F* = 52.427, *p* < 0.001 for FBN-ARO-KO; *F* = 341.915, *p* < 0.001 for GCI; *F* = 32.038, *p* < 0.001 for FBN-ARO-KO and GCI interaction; *p* < 0.001, *n* = 4, two-way ANOVA). In contrast, FBN-ARO-KO+GCI mice displayed a pronounced reduction of astrocytic GLT-1 levels compared with FLOX+GCI mice (*p* < 0.001, *n* = 4, two-way ANOVA). To further confirm the GLT-1 results, we performed Western blot analysis. Similar results were obtained to the IHC analysis, with GLT-1 protein levels significantly decreased in hippocampal lysate from FBN-ARO-KO+GCI mice than FLOX+GCI mice (Fig. 5*D*; *F* = 14.85, *p* = 0.006 for FBN-ARO-KO; *F* = 20.495, *p* < 0.001 for GCI; *F* = 10.067, *p* = 0.011 for FBN-ARO-KO and GCI interaction; *n* = 6 or 7, *p* = 0.026, two-way ANOVA). These results support our behavioral and transcriptomic data and suggest that neuron-derived E2 is critical for hippocampal astrocyte activation and upregulation of neuroprotective astrocyte-derived neurotrophins and GLT-1 after GCI.

**Figure 6.**
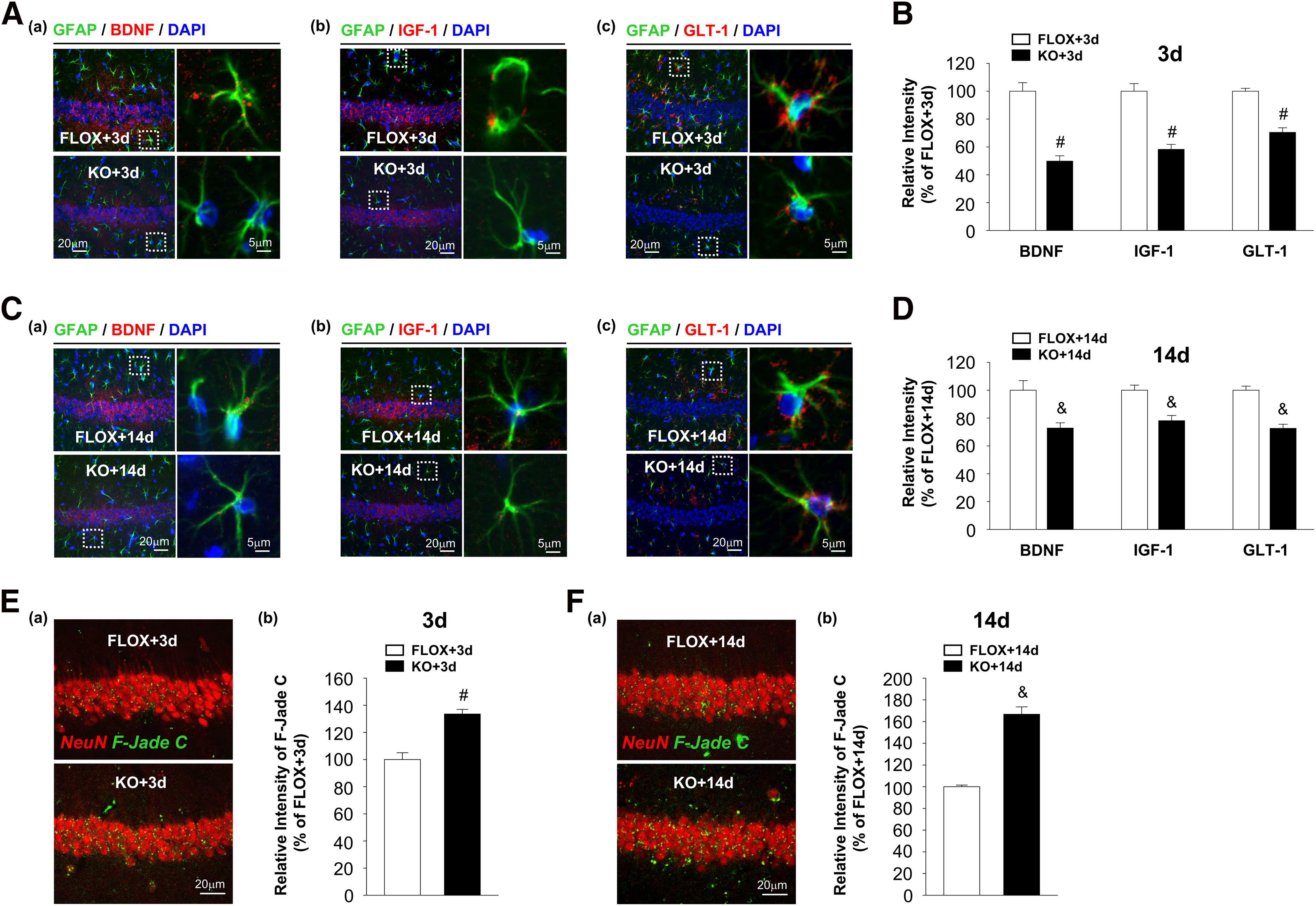
Depletion of neuronal E2 leads to compromised astrocyte functions and enhanced neuronal damage at 3 and 14 d after GCI in ovariectomized female FBN-ARO-KO mice. ***A***, ***B***, Alterations of astrocyte-derived BDNF (***Aa***), IGF-1 (***Ab***), and GLT-1 (***Ac***) in hippocampal CA1 region at 3 d after GCI were examined by IHC analysis. Intensities of astrocytic BDNF, IGF-1, and GLT-1 were quantitatively analyzed (***B***). ***C***, ***D***, BDNF (***Ca***), IGF-1 (***Cb***), and GLT-1 (***Cc***) expressions in astrocytes of hippocampal CA1 at 14 d after GCI were examined by IHC, and their relative changes in FBN-ARO-KO mice versus FLOX+GCI mice were quantified (***D***). ***E***, ***F***, Neuronal damage in hippocampal CA1 region at 3 d (***Ea***) and 14 d (***Fa***) after GCI was evaluated by NeuN and F-Jade C double staining. Relative intensities of F-Jade C at 3d (***Eb***) and 14d (***Fb***) were quantitatively analyzed. Values are mean ± SEM of determinations from each group. *N* = 4 or 5. ^#^*p* < 0.05 versus FLOX + 3 d. ^&^*p* < 0.05 versus FLOX + 14 d.

To examine whether these astrocyte-derived factors have similar alterations at other stages after GCI, we also evaluated BDNF, IGF-1, and GLT-1 levels in hippocampal astrocytes at 3 d (Fig. 6*A*) and 14 d (Fig. 6*C*) after GCI with double IHC analysis for GFAP and each factor. Interestingly, BDNF, IGF-1, and GLT-1 expression in FBN-ARO-KO+GCI astrocytes was also robustly decreased at both of these two time points compared with FLOX+GCI mice (Fig. 6*B*, 3 d; *t* = 6.936, *p* < 0.001 for BDNF; *t* = 6.392, *p* < 0.001 for IGF-1; *t* = 7.559, *p* < 0.001 for GLT-1; *n* = 4, unpaired *t* test; Fig. 6*D*, 14 d; *t* = 3.461, *p* = 0.013 for BDNF; *t* = 4.208, *p* = 0.006 for IGF-1; *t* = 6.636, *p* < 0.001 for GLT-1; *n* = 4, unpaired *t* test). These results indicate that astrocyte dysfunction occurs at early stage of GCI injury along with the diminished astrocyte activation and may cause persistent ischemic brain damage. Therefore, hippocampal neuronal injuries at these two stages were evaluated with F-Jade C and NeuN costaining (Fig. 6*E*,*F*). As expected, we observed elevated F-Jade C intensity in FBN-ARO-KO hippocampus at 3 d after GCI compared with the FLOX+GCI (*t* = −5.513, *p* = 0.001, *n* = 4, unpaired *t* test), which was even more enhanced at 14 d (*t* = −9.504, *p* < 0.001, *n* = 4, unpaired *t* test). These findings demonstrate that compromised astrocyte function after loss of neuron-derived E2 contributes to enhanced ischemic brain injury.

### Male FBN-ARO-KO mice exhibit similar deficits in astrocyte activation as observed in ovariectomized female mice after GCI

**Figure 7.**
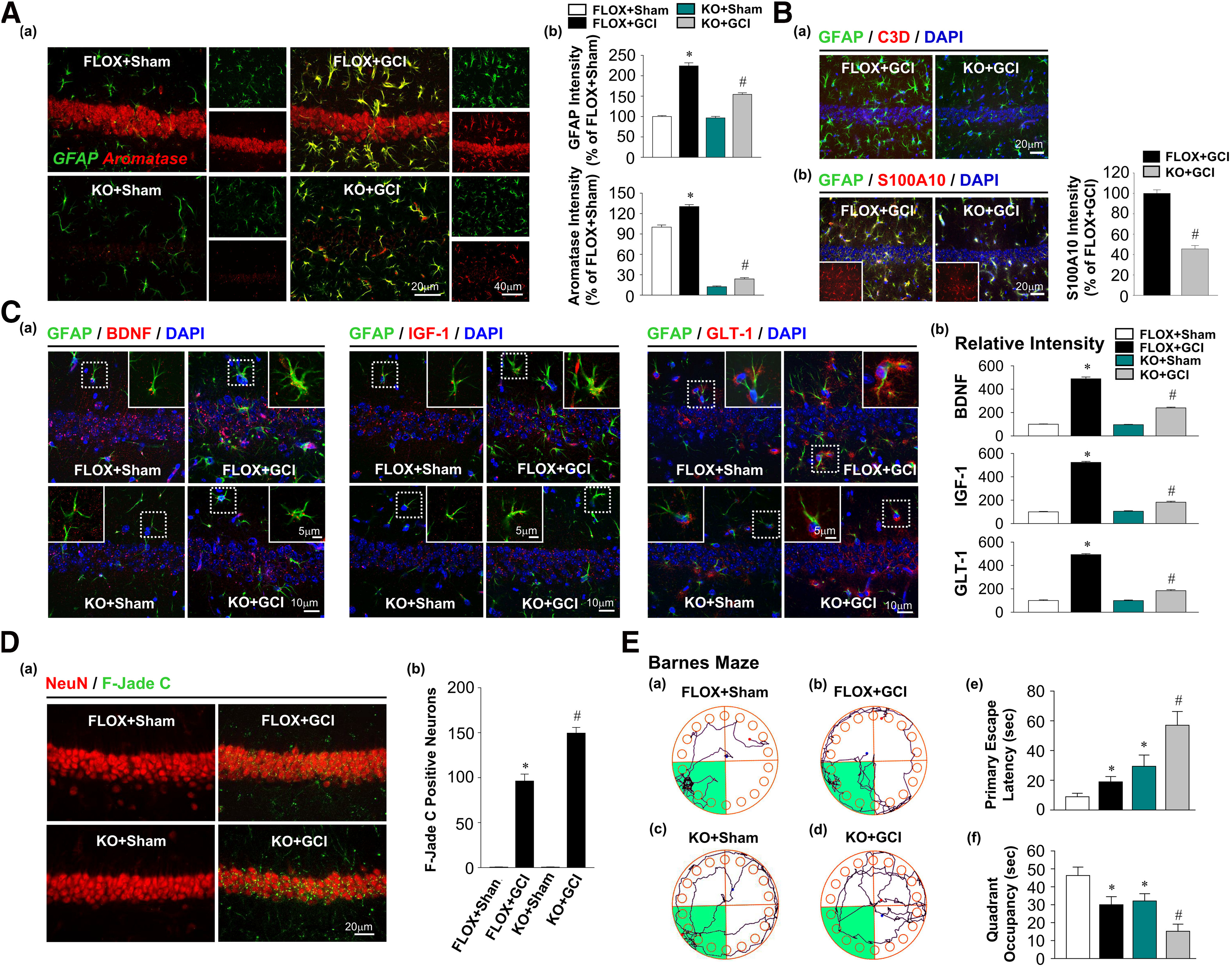
Male FBN-ARO-KO mice exhibit strong neurodegeneration and cognitive impairment 7 d after GCI injury. ***Aa***, Astrocyte activation and aromatization in hippocampal CA1 region were examined by GFAP and aromatase double staining. ***Ab***, GFAP levels in each group were quantified as a parameter of astrocyte reactivity after GCI injury. Total aromatase levels in hippocampal CA1 region were further measured. ***B***, Astrocyte A1 and A2 phenotypes after GCI were examined by IHC analysis with the markers of C3D (***Ba***) and S100A10 (***Bb***), respectively. ***Ca***, BDNF, IGF-1, and GLT-1 production in astrocytes following the altered astrocyte activation was determined by IHC analysis. ***Cb***, Their relative changes of intensities in astrocytes were quantified. ***Da***, Representative double staining for NeuN and F-Jade C to evaluate neuronal degeneration in each group. ***Db***, F-Jade C-positive neurons were counted. ***E***, Spatial reference memory was assessed by Barnes Maze behavioral test. ***Ea-Ed***, Tracking plots in probe trial. Primary escape latency (***Ee***) and quadrant occupancy (***Ef***) of animals from each group in probe trial were recorded. Values are mean ± SEM of determinations from each group. *N* = 4 for ***A-C***; *N* = 8-11 for ***D***. *p < 0.05 versus FLOX+Sham. ^#^*p* < 0.05 versus FLOX+GCI.

To address whether neuronal-derived E2 has similar functions in males, we next examined astrocyte reactivity, astrocyte polarization, hippocampal neuronal damage, and hippocampal-dependent cognitive function in intact male FBN-ARO-KO mice at 7 d after GCI. Double staining of aromatase and GFAP revealed that aromatase was specifically localized in hippocampal CA1 neurons in FLOX+Sham mice, which was profoundly decreased in FBN-ARO-KO+Sham mice (Fig. 7*Aa*). Aromatase was not detected in the resting astrocytes from either group. In contrast, aromatase was strongly induced in hypertrophic astrocytes of FLOX+GCI mice. Furthermore, the FBN-ARO-KO+GCI astrocytes appeared significantly less hypertrophic compared with astrocytes from FLOX+GCI mice, as manifested by robustly attenuated GFAP expression in FBN-ARO-KO+GCI mice (*F* = 60.266, *p* < 0.001 for FBN-ARO-KO; *F* = 370.573, *p* < 0.001 for GCI; *F* = 49.677, *p* < 0.001 for FBN-ARO-KO and GCI interaction; *p* < 0.001, *n* = 4, two-way ANOVA; Fig. 7*Ab*). In addition, aromatase expression in FBN-ARO-KO+GCI mice was strongly decreased compared with FLOX+GCI mice (*F* = 1624.577, *p* < 0.001 for FBN-ARO-KO; *F* = 74.823, *p* < 0.001 for GCI; *F* = 15.289, *p* = 0.002 for FBN-ARO-KO and GCI interaction; *p* < 0.001, *n* = 4, two-way ANOVA; Fig. 7*Ab*). These findings suggest that similar to the results in ovariectomized female mice, there is diminished astrocyte activation and aromatization in male FBN-ARO-KO mice after GCI.

Next, we determined the reactive astrocyte phenotype by double IHC analysis for GFAP and either the A1 marker C3D or the A2 marker S100A10. The results revealed that there was no detectable C3D expression in either the FLOX+GCI or FBN-ARO-KO+GCI hippocampus at 7 d after GCI, suggesting that no “A1” astrocytes were induced, a finding similar to that observed in ovariectomized female mice (Fig. 7*Ba*). However, double IHC revealed a pronounced decrease in the astrocyte “A2” marker S100A10 in the hippocampus of FBN-ARO-KO+GCI male mice compared with FLOX+GCI male mice (*t* = 11.373, *p* < 0.001, *n* = 4, unpaired *t* test; Fig. 7*Bb*). We next assessed expression of astrocytic neurotrophins and glutamate transporter GLT-1. Similar to the results in ovariectomized female mice, double IHC analysis confirmed pronounced reductions in both BDNF (*F* = 212.013, *p* < 0.001 for FBN-ARO-KO; *F* = 939.454, *p* < 0.001 for GCI; *F* = 199.614, *p* < 0.001 for FBN-ARO-KO and GCI interaction; *p* < 0.001, *n* = 4, two-way ANOVA) and IGF-1 (*F* = 614.323, *p* < 0.001 for FBN-ARO-KO; *F* = 1361.41, *p* < 0.001 for GCI; *F* = 650.955, *p* < 0.001 for FBN-ARO-KO and GCI interaction; *p* < 0.001, *n* = 4, two-way ANOVA) levels in hippocampal astrocytes of male FBN-ARO-KO+GCI mice compared with male FLOX+GCI mice (Fig. 7*C*). A significant reduction in astrocytic GLT-1-immunoreactive protein levels was also observed in FBN-ARO-KO+GCI mice compared with male FLOX+GCI mice (*F* = 472.203, *p* < 0.001 for FBN-ARO-KO; *F* = 1126.799, *p* < 0.001 for GCI; *F* = 468.854, *p* < 0.001 for FBN-ARO-KO and GCI interaction; *p* < 0.001, *n* = 4, two-way ANOVA; Fig. 7*C*). Further examination for neuronal damage in hippocampal CA1 region by F-Jade C staining revealed that the number of degenerating neurons in male FBN-ARO-KO+GCI mice was significantly increased compared with male FLOX+GCI mice (Fig. 7*D*; *F* = 28.6, *p* < 0.001 for FBN-ARO-KO; *F* = 604.186, *p* < 0.001 for GCI; *F* = 28.6, *p* < 0.001 for FBN-ARO-KO and GCI interaction; *p* = 0.002, *n* = 4, two-way ANOVA). Finally, examination of hippocampal-dependent spatial reference memory by Barnes Maze Test revealed that male FBN-ARO-KO mice exhibited an enhanced memory deficit compared with male FLOX mice at 7 d after GCI (Fig. 7*E*), as evidenced by an increased escape latency (*F* = 18.884, *p* < 0.001 for FBN-ARO-KO; *F* = 7.801, *p* = 0.009 for GCI; *F* = 4.702, *p* = 0.021 for FBN-ARO-KO and GCI interaction; *p* = 0.007, *n* = 11, two-way ANOVA) and decreased exploring time (*F* = 10.831, *p* = 0.002 for FBN-ARO-KO; *F* = 14.217, *p* < 0.001 for GCI; *F* = 3.92, *p* = 0.045 for FBN-ARO-KO and GCI interaction; *p* = 0.023, *n* = 11, two-way ANOVA) in the target quadrant.

**Figure 8.**
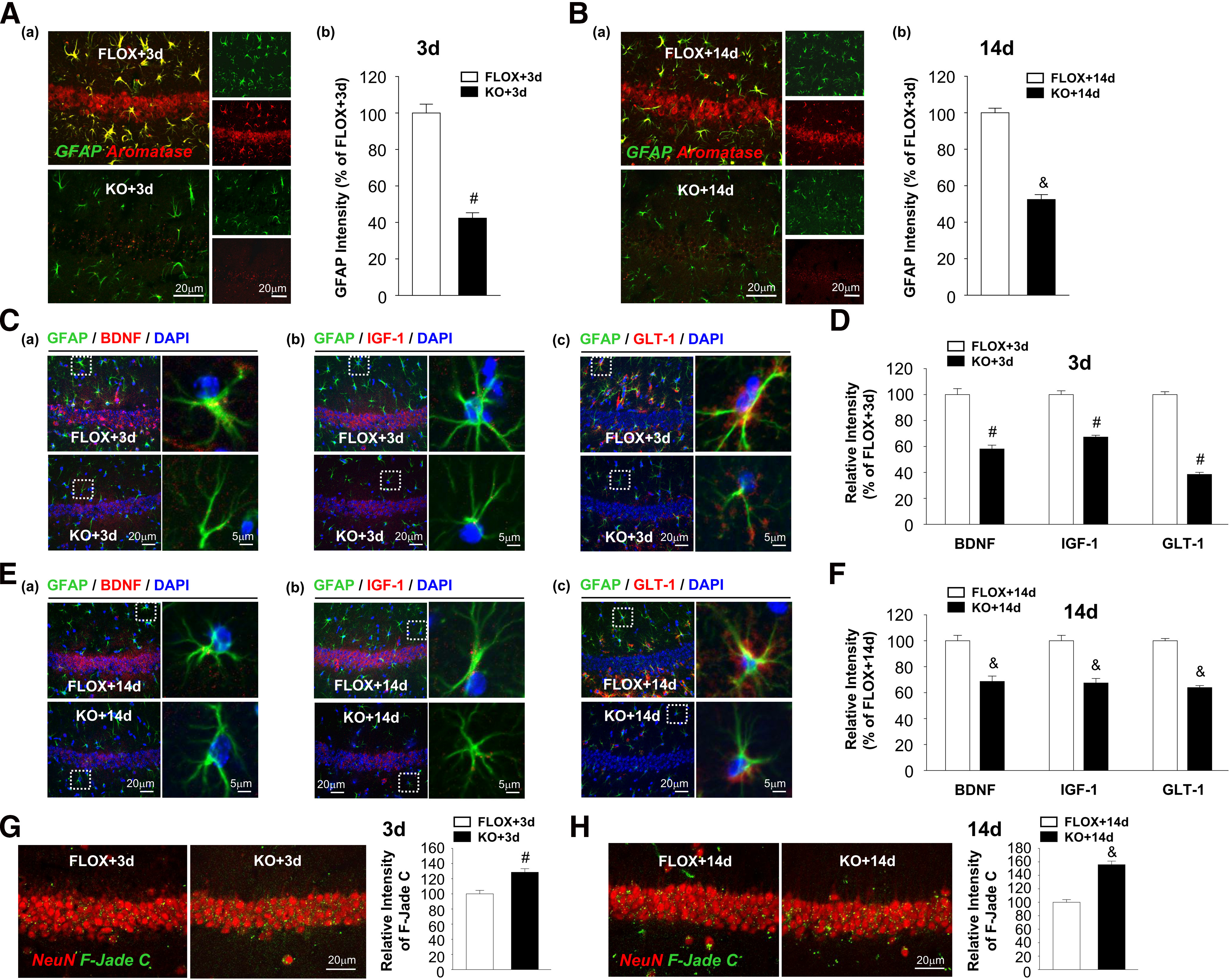
Male FBN-ARO-KO mice display attenuated astrocyte activation, compromised astrocyte functions, and worse neuronal damage at 3 and 14 d after GCI. ***A***, ***B***, GFAP and aromatase double staining to examine astrocyte activation and astrocytic aromatase induction at 3 d (***Aa***) and 14 d (***Ba***) after GCI. Relative changes of GFAP intensities at 3d (***Ab***) and 14d (***Bb***) were quantified. ***C***, Astrocyte-derived BDNF (***Ca***), IGF-1 (***Cb***), and GLT-1 (***Cc***) levels at 3 d after GCI were explored by IHC analysis. ***D***, Their relative intensities in KO + 3 d mice versus FLOX + 3 d mice. ***E***, ***F***, BDNF (***Ea***), IGF-1 (***Eb***), and GLT-1 (***Ec***) levels in hippocampal astrocytes at 14 d after GCI were determined with IHC, and expressed as relative changes of KO + 14 d mice compared with FLOX + 14 d mice (***F***). ***G***, ***H***, Neuronal damage in hippocampal CA1 region at 3 d (***G***) and 14 d (***H***) after GCI was assessed by NeuN and F-Jade C double staining. Values are mean ± SEM of determinations from each group. *N* = 4. ^#^*p* < 0.05 versus FLOX + 3 d. ^&^*p* < 0.05 versus FLOX + 14 d.

Similarly, as observed in females, we found strongly decreased GFAP expression and aromatase induction in astrocytes in male FBN-ARO-KO+GCI mice at both 3 d (Fig. 8*A*; *t* = 10.207, *p* < 0.001, *n* = 4, unpaired *t* test) and 14 d (Fig. 8*B*; *t* = 13.037, *p* < 0.001, *n* = 4, unpaired *t* test) after GCI, indicative of diminished astrocyte activation and aromatization. Further investigation for astrocyte-derived BDNF, IGF-1, and GLT-1 in the hippocampal CA1 region also showed significant downregulation in male FBN-ARO-KO mice at both 3 d (Fig. 8*C*,*D*; *t* = 7.73, *p* < 0.001 for BDNF; *t* = 10.185, *p* < 0.001 for IGF-1; *t* = 22.435, *p* < 0.001 for GLT-1; *n* = 4, unpaired *t* test) and 14 d (Fig. 8*E*,*F*; *t* = 5.356, *p* = 0.002 for BDNF; *t* = 6.008, *p* < 0.001 for IGF-1; *t* = 15.831, *p* < 0.001 for GLT-1; *n* = 4, unpaired *t* test) following GCI reperfusion. Correspondingly, hippocampal neuronal damage was worse in FBN-ARO-KO+GCI mice at these time points, as evidenced by enhanced F-Jade C intensity in hippocampal CA1 neurons (Fig. 8*G*, 3 d; *t* = −4.381, *p* = 0.005, *n* = 4, unpaired *t* test; Fig. 8*H*, 14 d; *t* = −8.535, *p* < 0.001, *n* = 4, unpaired *t* test).

### Enhanced neuronal FGF2 signaling mediates the diminishment in astrocyte activation in FBN-ARO-KO mice after GCI

**Figure 9.**
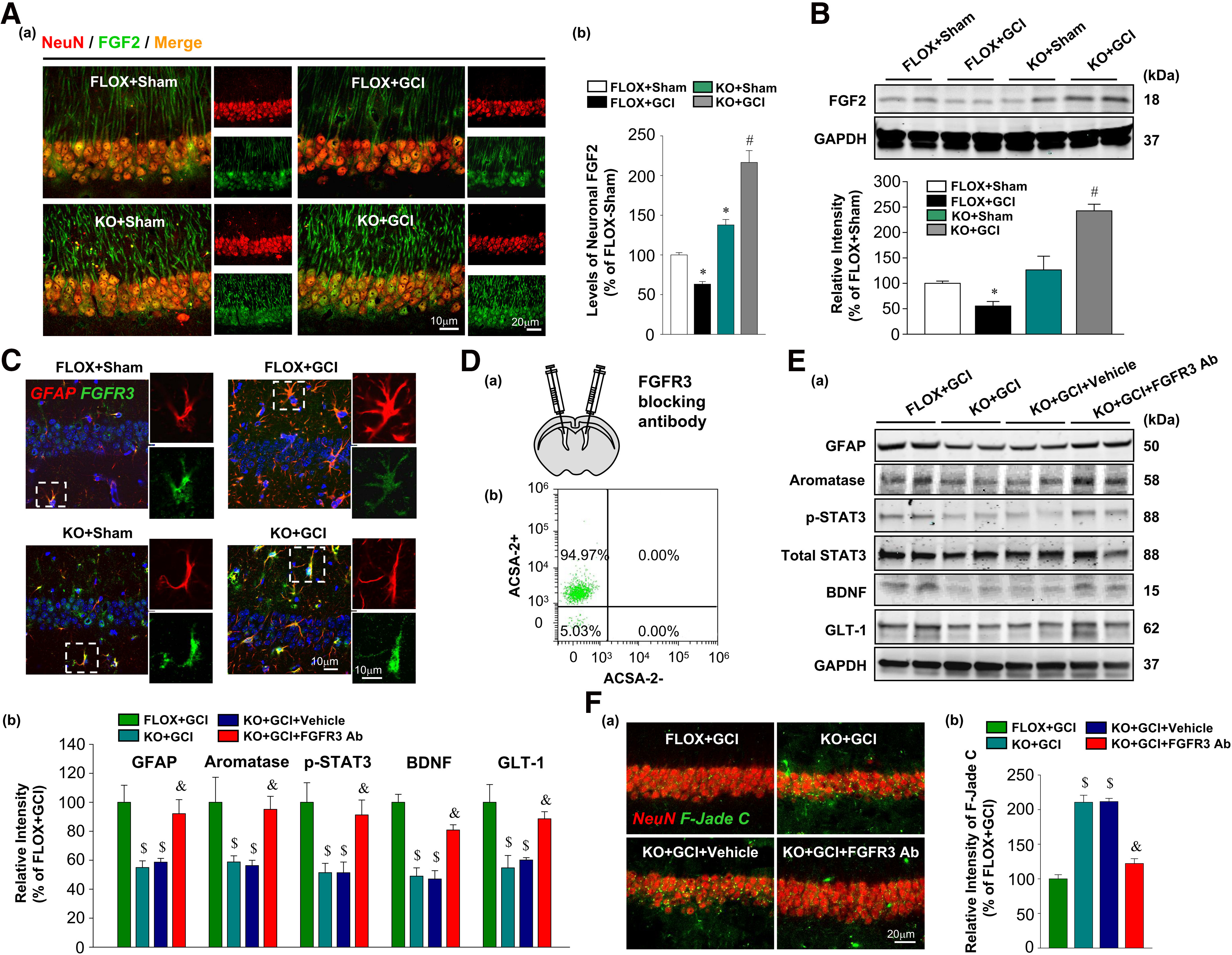
Evidence that upregulation of neuronal FGF2 signaling mediates decreased astrocyte activation in ovariectomized female FBN-ARO-KO mice after GCI. ***Aa***, Representative image of FGF2 and NeuN double staining in hippocampal CA1 region. ***Ab***, Quantitative analysis of neuronal FGF2 levels in each group (*N* = 4 or 5). ***B***, Neuronal FGF2 alterations in hippocampus were confirmed by Western blotting analysis (*N* = 3). ***C***, Representative images of FGFR3 and GFAP double staining showing changes of FGFR3 expression in hippocampal CA1 astrocytes (*N* = 4 or 5). ***Da***, Schematic illustration of FGFR3 neutralization by bilateral intracerebroventricular injection of FGFR3 blocking antibody in FBN-ARO-KO GCI mice. ***Db***, The purity of isolated astrocytes from the ischemic brains was evaluated by flow cytometry analysis. ***E***, To examine the effects of FGFR3 neutralization on astrocyte reactivity and functional restoration in FBN-ARO-KO mice after GCI, levels of GFAP, p-STAT3, BDNF, and GLT-1 in purified astrocytes were determined by Western blotting (***Ea***), and further quantitatively analyzed (***Eb***) (*N* = 3). ***Fa***, IHC analysis for neuronal degeneration by F-Jade C and NeuN double staining in hippocampal CA1 region. ***Fb***, Quantification of F-Jade C levels in CA1 pyramidal neurons, which was presented as relative intensity of each group versus FLOX+GCI (*N* = 4 or 5). Values are mean ± SEM of determinations from each group. *p < 0.05 versus FLOX+Sham. ^#^*p* < 0.05 versus FLOX+GCI. ^$^*p* < 0.05 versus FLOX+GCI. ^&^*p* < 0.05 versus KO+GCI.

Next, we explored the potential mechanisms underlying how neuronal-derived E2 regulates reactive astrocyte induction after GCI. We focused on neuronal FGF2 signaling, as it has been previously shown to suppress astrocyte activation (Kang et al., 2014; Y. Zhang et al., 2017), and our RNA-Seq results revealed that the FGF2 transcript was markedly increased in the hippocampus of FBN-ARO-KO+GCI mice versus FLOX+GCI mice. This led us to hypothesize that FGF2 signaling might be a key factor that mediates the suppression of reactive astrogliosis in FBN-ARO-KO mice following GCI injury. First, we examined FGF2 levels in hippocampal CA1 neurons of ovariectomized female mice by double staining with FGF2 and NeuN. We found a significant increase of neuronal FGF2 in FBN-ARO-KO+GCI mice compared with the other groups, especially FLOX+GCI mice (Fig. 9*A*; *F* = 126.745, *p* < 0.001 for FBN-ARO-KO; *F* = 6.092, *p* = 0.025 for GCI; *F* = 46.305, *p* < 0.001 for FBN-ARO-KO and GCI interaction; *p* = 0.008, *n* = 5, two-way ANOVA). Conversely, FLOX+GCI mice actually displayed a further reduction in neuronal FGF2 level compared with FLOX+Sham (*p* < 0.001, *n* = 5, two-way ANOVA), which is consistent with previous reports that reduced neuronal FGF2 facilitates reactive astrogliosis (Y. Zhang et al., 2017). Western blot was further conducted to validate hippocampal FGF2 alterations, which confirmed the robust increase of FGF2 protein levels in FBN-ARO-KO+GCI hippocampus compared with the other groups (Fig. 9*B*). To investigate the interaction of this increased neuronal FGF2 with neighboring astrocytes, we next examined the expression of its major receptor FGFR3 in hippocampal astrocytes by IHC analysis (Fig. 9*C*). Interestingly, we observed weak intensity of FGFR3 in the reactive astrocytes of FLOX+GCI mice, corresponding to the downregulated neuronal FGF2 level. In contrast, strong FGFR3 was expressed in both the resting astrocytes of noninjured sham groups and the less activated astrocytes of FBN-ARO-KO+GCI mice, suggesting that increased FGF2 signaling in FBN-ARO-KO+GCI mice might contribute to the diminished astrogliosis observed in these mice after GCI.

To confirm this possibility, we used a previously established FGFR3 antibody neutralizing approach to block cerebral FGF2 signaling in ovariectomized female FBN-ARO-KO+GCI mice (Y. Zhang et al., 2017). FGFR3 antibody was bilaterally infused by intracerebroventricular injection at the time of GCI reperfusion (Fig. 9*Da*). To determine astrocyte reactivity, aromatization, and the specific expression of astrocyte neurotrophic/neuroprotective factors following FGFR3 blockage, we isolated astrocytes from the ischemic brains at 7 d after GCI and determined a 94.97% astrocyte purity through flow cytometry (Fig. 9*Db*). Western blot analysis was subsequently conducted with protein lysate of the purified astrocytes (Fig. 9*E*). It was shown that GFAP levels in purified astrocytes isolated from FBN-ARO-KO+GCI+FGFR3 Ab mice were elevated to ∼92.1% of FLOX+GCI levels (*F* = 11.027, *p* = 0.011 for FBN-ARO-KO; *F* = 9.448, *p* = 0.028 for FGFR3-Blockage; *F* = 8.629, *p* = 0.032 for FBN-ARO-KO and FGFR3-Blockage interaction; *p* = 0.026, *n* = 3, two-way ANOVA), indicating an almost complete rescue of reactive astrogliosis in FBN-ARO-KO+GCI mice after blocking FGF2 signaling. We also observed pronounced aromatase induction in FBN-ARO-KO+GCI astrocytes, to levels ∼95.1% of FLOX+GCI mice (*F* = 12.014, *p* = 0.004 for FBN-ARO-KO; *F* = 10.641, *p* = 0.013 for FGFR3-Blockage; *F* = 8.846, *p* = 0.021 for FBN-ARO-KO and FGFR3-Blockage interaction; *p* = 0.016, *n* = 3, two-way ANOVA). Furthermore, examination of p-STAT3, a hallmark of neuroprotective astrocyte phenotype (Burda and Sofroniew, 2014; Tyzack et al., 2014), revealed that pSTAT3 levels in the purified astrocytes isolated from FBN-ARO-KO+GCI+FGFR3 Ab mice were elevated to ∼91.2% of FLOX+GCI levels (*F* = 9.631, *p* = 0.015 for FBN-ARO-KO; *F* = 7.662, *p* = 0.023 for FGFR3-Blockage; *F* = 6.328, *p* = 0.031 for FBN-ARO-KO and FGFR3-Blockage interaction; *p* = 0.03, *n* = 3, two-way ANOVA). These results demonstrate that the attenuated astrocyte activation and aromatase expression in FBN-ARO-KO+GCI mice were effectively reinstated by blocking FGF2 signaling. Further study by Western blot analysis revealed that FGFR3 neutralization robustly rescued both BDNF (*F* = 67.69, *p* < 0.001 for FBN-ARO-KO; *F* = 14.864, *p* = 0.005 for FGFR3-Blockage; *F* = 12.612, *p* = 0.011 for FBN-ARO-KO and FGFR3-Blockage interaction; *p* = 0.009, *n* = 3, two-way ANOVA) and GLT-1 (*F* = 14.848, *p* = 0.005 for FBN-ARO-KO; *F* = 14.562, *p* = 0.006 for FGFR3-Blockage; *F* = 10.367, *p* = 0.025 for FBN-ARO-KO and FGFR3-Blockage interaction; *p* = 0.026, *n* = 3, two-way ANOVA) expression in purified astrocytes isolated from FBN-ARO-KO+GCI+FGFR3 Ab mice. Collectively, these findings demonstrate that neuroprotective astrocyte activation and functions were significantly restored after FGFR3 neutralization in FBN-ARO-KO mice after GCI.

To determine whether reinstatement of the beneficial astrocyte response after FGFR3 neutralization can lead to reduced neuronal damage in FBN-ARO-KO+GCI mice, we next performed F-Jade C and NeuN costaining. The results showed that F-Jade C intensity in hippocampal CA1 neurons was significantly attenuated in FBN-ARO-KO+GCI mice by FGFR3 neutralization, indicating decreased hippocampal neuronal degeneration (Fig. 9*Fa*,*b*; *F* = 169.025, *p* < 0.001 for FBN-ARO-KO; *F* = 75.747, *p* < 0.001 for FGFR3-Blockage; *F* = 71.694, *p* < 0.001 for FBN-ARO-KO and FGFR3-Blockage interaction; *p* < 0.001, *n* = 4, two-way ANOVA). Together, our results indicate that neuronal-derived E2 regulation of neuroprotective astrogliosis after GCI is due, at least in part, to suppression of neuronal FGF2 signaling.

### *In vivo* exogenous E2 replacement reverses astrocyte dysfunction and neuronal damage in ovariectomized female FBN-ARO-KO mice

**Figure 10.**
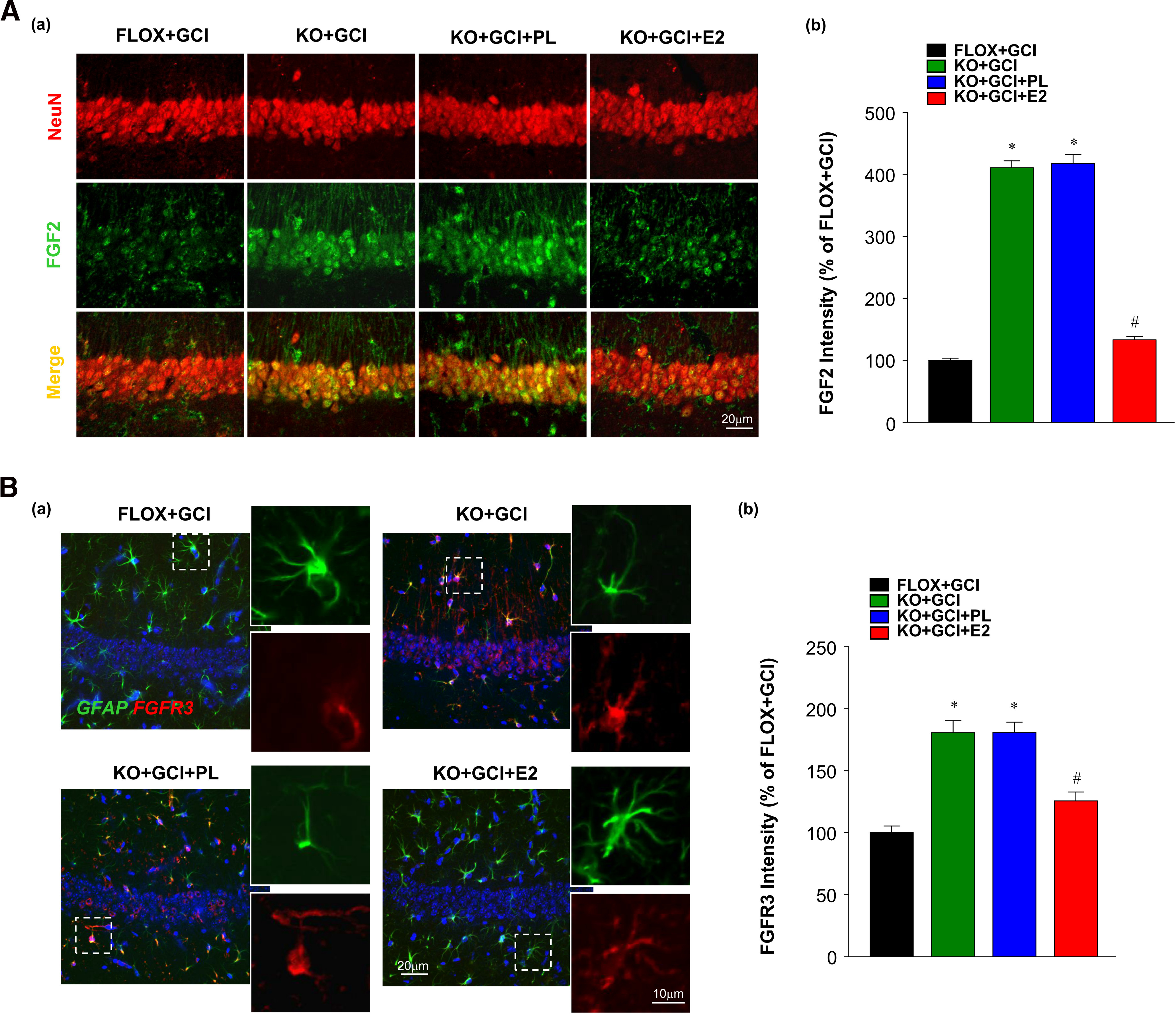
Exogenous E2 replacement decreases hippocampal FGF2 signaling in ovariectomized female FBN-ARO-KO mice. ***Aa***, Effect of exogenous E2 replacement on neuronal FGF2 expression in FBN-ARO-KO mice was examined by NeuN and FGF2 double staining. ***Ab***, Relative neuronal FGF2 intensities after E2 treatment were quantitatively analyzed. ***Ba***, Levels of the major FGF2 receptor FGFR3 in hippocampal astrocytes were determined by GFAP and FGFR3 staining. ***Bb***, FGFR3 relative intensities in FBN-ARO-KO mice after E2 rescue were quantified. Values are mean ± SEM of determinations from each group. *N* = 4. *p < 0.05 versus FLOX+GCI. ^#^*p* < 0.05 versus KO+GCI.

We next performed an *in vivo* exogenous E2 rescue experiment to determine whether the neuronal defects in FBN-ARO-KO+GCI mice could be prevented when brain equivalent E2 was reinstated in FBN-ARO-KO+GCI mice. Exogenous E2 was administered in ovariectomized female FBN-ARO-KO mice by minipumps, with a dose that has been previously demonstrated to fully restore the hippocampal E2 levels in FBN-ARO-KO mice (Lu et al., 2019). First, the effect of E2 treatment on hippocampal FGF2 signaling was explored. Double staining for NeuN and FGF2 revealed that the strongly increased neuronal FGF2 expression in FBN-ARO-KO+GCI mice was significantly repressed after E2 rescue to a level close to FLOX+GCI mice (Fig. 10*A*; *F* = 713.65, *p* < 0.001 for FBN-ARO-KO; *F* = 466.031, *p* < 0.001 for E2 treatment; *F* = 421.49, *p* < 0.001 for FBN-ARO-KO and E2 treatment interaction; *p* < 0.001, *n* = 4, two-way ANOVA). No alterations were found with placebo (vehicle) treatment (*p* = 0.723, *n* = 4, two-way ANOVA). The expression of the major FGF2 receptor, FGFR3 in hippocampal astrocytes was further examined by GFAP and FGFR3 double staining (Fig. 10*B*). The results show that FGFR3, which was weakly expressed in the processes of reactive astrocytes in the FLOX+GCI hippocampal CA1 region, was robustly elevated in the whole cell body of less activated astrocytes in FBN-ARO-KO+GCI mice (*F* = 64.532, *p* < 0.001 for FBN-ARO-KO; *F* = 17.246, *p* = 0.001 for E2 treatment; *F* = 16.287, *p* = 0.001 for FBN-ARO-KO and E2 treatment interaction; *p* < 0.001, *n* = 4, two-way ANOVA). In contrast, E2 treatment significantly decreased the astrocytic FGFR3 level (*p* = 0.003, *n* = 4, two-way ANOVA) compared with placebo (vehicle) treatment, confirming the inhibitory role of E2 on FGF2 signaling.

**Figure 11.**
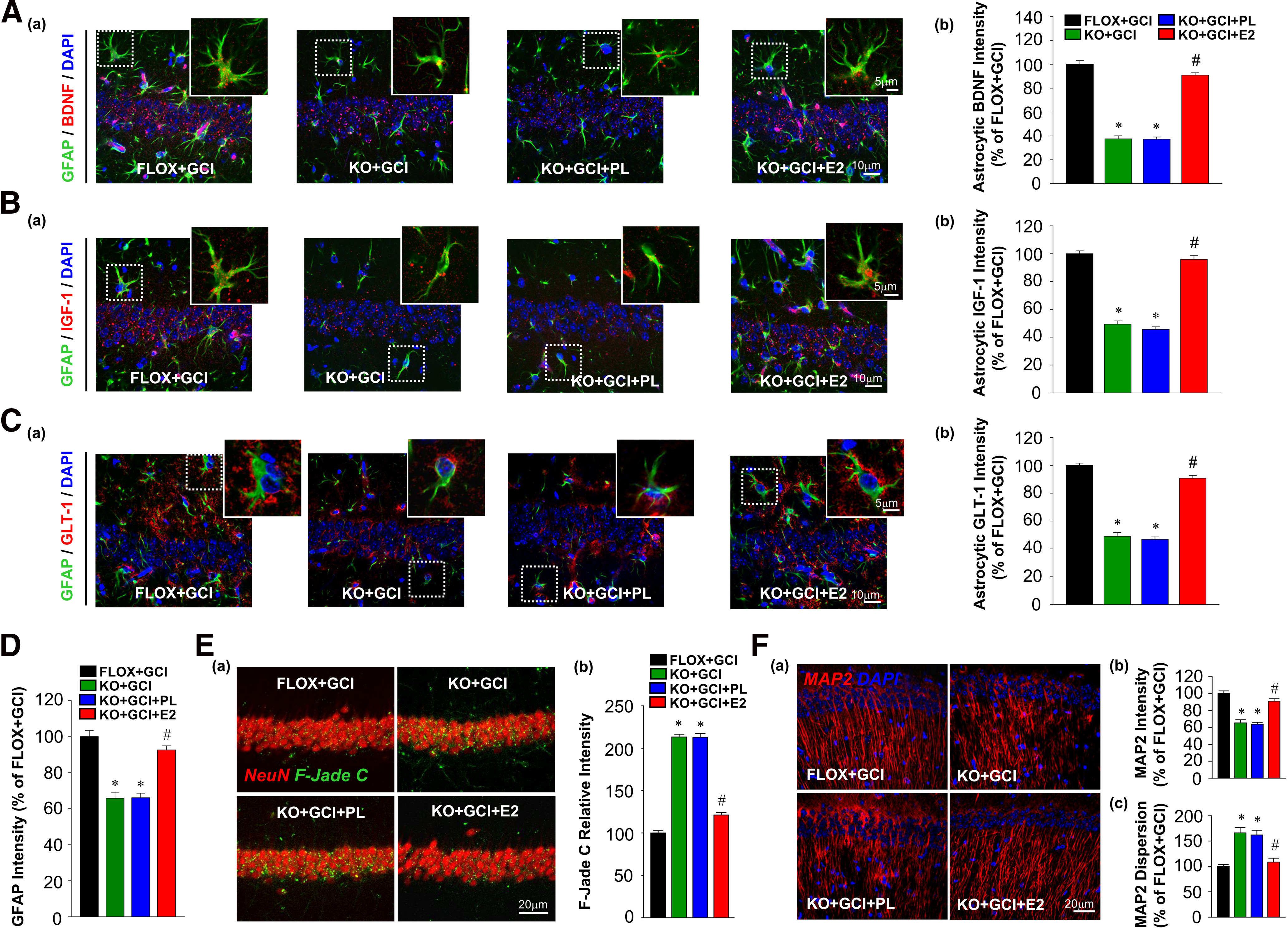
Exogenous E2 replacement reverses the protective astrocyte dysfunction and neuronal damage in ovariectomized female FBN-ARO-KO mice. ***A–C***, Representative images of BDNF (***Aa***), IGF-1 (***Ba***), and GLT-1 (***Ca***) with GFAP double immunostaining to determine the functional outcome of astrocytes in hippocampal CA1 subregion of FBN-ARO-KO GCI mice after 7 d exogenous E2 replacement. Relative intensities of astrocyte-derived BDNF (***Ab***), IGF-1 (***Bb***) and GLT-1 (***Cb***) were quantitatively analyzed. ***D***, GFAP intensities were analyzed to determine the astrocyte reactivity in FBN-ARO-KO GCI mice following E2 rescue. ***Ea***, IHC analysis for neuronal damage by examining F-Jade C levels in pyramidal neurons of hippocampal CA1 region. ***Eb***, Numbers of F-Jade C-positive neurons per 250 µm in each group were counted. ***Fa***, Dendritic morphologic analysis by MAP2 immunostaining to evaluate the structural integrity of hippocampal CA1 neurons. ***Fb***, MAP2 intensity and dispersion were further quantified. Values are mean ± SEM of determinations from each group. *N* = 4. *p < 0.05 versus FLOX+GCI. ^#^*p* < 0.05 versus KO+GCI.

Next, we further examined the effects of E2 rescue on astrocyte functions in FBN-ARO-KO+GCI mice. As shown in Figure 11*A-C*, IHC analysis showed that exogenous E2 replacement was able to rescue astrocyte-derived BDNF (Fig. 11*A*; *F* = 232.568, *p* < 0.001 for FBN-ARO-KO; *F* = 129.903, *p* < 0.001 for E2 treatment; *F* = 118.67, *p* < 0.001 for FBN-ARO-KO and E2 treatment interaction; *p* < 0.001, *n* = 4, two-way ANOVA), IGF-1 (Fig. 11*B*; *F* = 233.13, *p* < 0.001 for FBN-ARO-KO; *F* = 175.496, *p* < 0.001 for E2 treatment; *F* = 122.551, *p* < 0.001 for FBN-ARO-KO and E2 treatment interaction; *p* < 0.001, *n* = 4, two-way ANOVA), and GLT-1 (Fig. 11*C*; *F* = 413.521, *p* < 0.001 for FBN-ARO-KO; *F* = 219.227, *p* < 0.001 for E2 treatment interaction; *F* = 129.554, *p* < 0.001 for FBN-ARO-KO and E2 treatment interaction; *p* < 0.001, *n* = 4, two-way ANOVA) in FBN-ARO-KO+GCI hippocampus to almost FLOX+GCI levels. No alterations were found with placebo (vehicle) treatment. To determine whether reactive astrogliosis was also reinstated after E2 treatment, we analyzed GFAP intensity in the hippocampal CA1 region (Fig. 11*D*). We found E2 replacement rescued GFAP levels in FBN-ARO-KO+GCI mice from 65.7% of FLOX+GCI mice to 92.6% (*F* = 92.543, *p* < 0.001 for FBN-ARO-KO; *F* = 43.672, *p* < 0.001 for E2 treatment; *F* = 18.127, *p* = 0.001 for FBN-ARO-KO and E2 treatment interaction; *p* < 0.001, *n* = 4, two-way ANOVA), while placebo treatment did not cause any changes (*p* = 0.943, *n* = 4, two-way ANOVA). Neuronal injury was next assessed by F-Jade C staining. The results in Figure 11*E* showed that the increased F-Jade C intensity in FBN-ARO-KO+GCI hippocampal CA1 neurons was robustly decreased by E2 replacement (*F* = 490.987, *p* < 0.001 for FBN-ARO-KO; *F* = 237.039, *p* < 0.001 for E2 treatment; *F* = 192.664, *p* < 0.001 for FBN-ARO-KO and E2 treatment interaction; *p* < 0.001, *n* = 4, two-way ANOVA). Neuronal structure was further examined by MAP2 staining (Fig. 11*F*), which revealed that the reduced MAP2 intensity and elevated MAP2 dispersion in FBN-ARO-KO+GCI mice were subsequently reversed by exogenous E2 treatment (MAP2 intensity, *F* = 98.423, *p* < 0.001 for FBN-ARO-KO; *F* = 39, *p* < 0.001 for E2 treatment; *F* = 24.143, *p* < 0.001 for FBN-ARO-KO and E2 treatment interaction; *p* < 0.001, *n* = 4, two-way ANOVA; MAP2 dispersion, *F* = 33.174, *p* < 0.001 for FBN-ARO-KO; *F* = 18.906, *p* < 0.001 for E2 treatment; *F* = 17.872, *p* < 0.001 for FBN-ARO-KO and E2 treatment interaction; *p* = 0.004, *n* = 4, two-way ANOVA).

**Figure 12.**
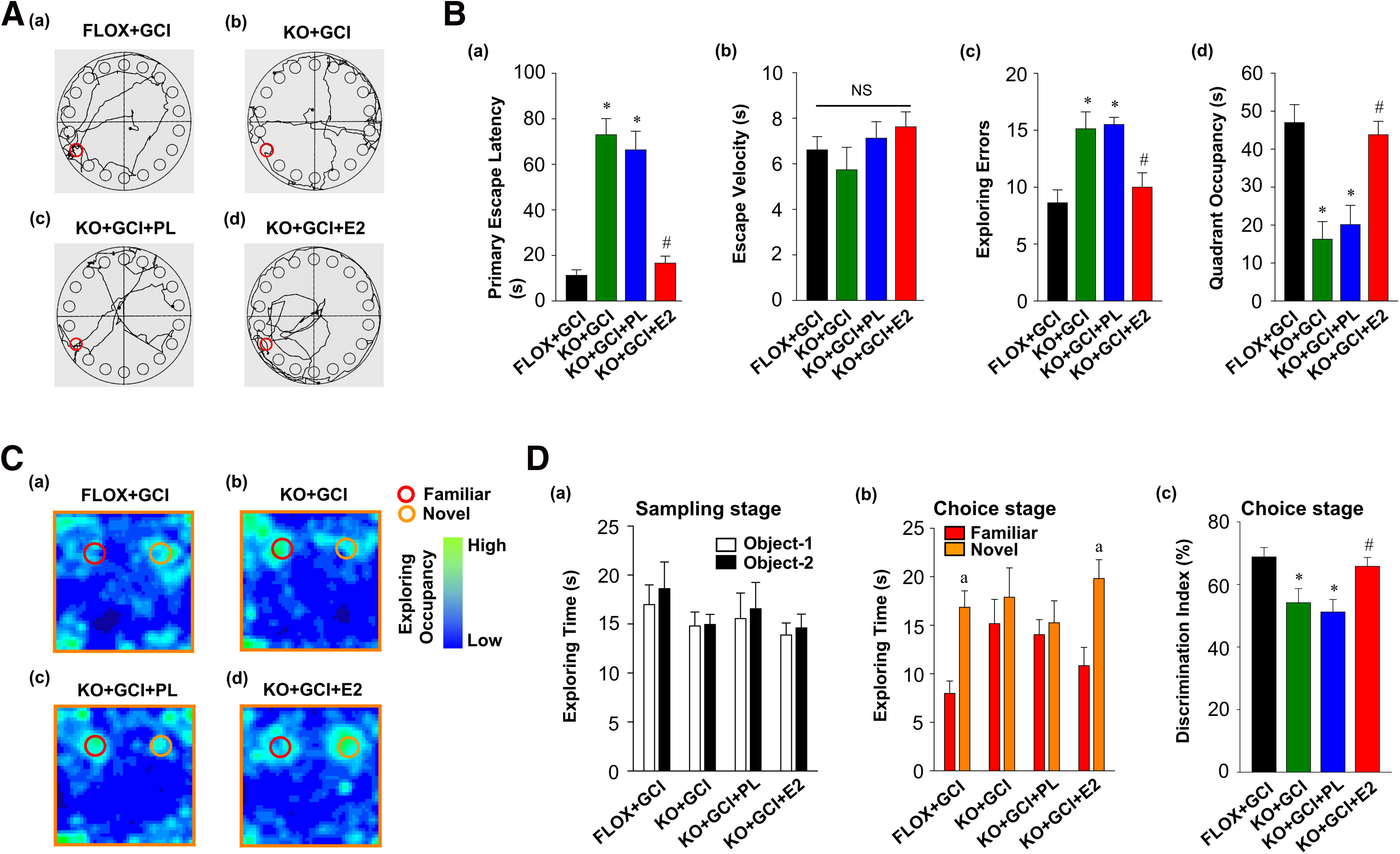
Hippocampal-dependent cognitive functions were preserved by exogenous E2 replacement in ovariectomized female FBN-ARO-KO mice after GCI injury. ***A***, Hippocampal-dependent spatial reference memory of FBN-ARO-KO mice, which were subjected to E2 rescue, was evaluated by Barnes Maze Test. ***Aa–Ad***, Tracking plot of the indicated groups in the probe trial. ***B***, Primary escape latency (***Ba***), escape velocity (***Bb***), exploring errors (***Bc***), and quadrant occupancy (***Bd***) were recorded. ***C***, Hippocampal-dependent recognition memory was subsequently examined by Novel Object Recognition Test. ***Ca***–***Cd***, The occupancy plot of each group in the choice stage. ***D***, Exploring time on two resemble objects in the sampling stage (***Da***), exploring time on familiar and novel objects, respectively, in the choice stage (***Db***) and the following discrimination index (***Dc***) were analyzed. Values are mean ± SEM of determinations from each group. *N* = 8. *p < 0.05 versus FLOX+GCI. ^#^*p* < 0.05 versus KO+GCI. ^a^*p* < 0.05 versus Familiar object. NS, no significant difference.

We next conducted the Barnes Maze Test to evaluate whether exogenous E2 replacement was able to rescue hippocampal-dependent spatial reference learning and memory in FBN-ARO-KO mice after GCI. As shown in Figure 12*A*, tracking plots in the probe trial revealed significantly improved performance on exploring the target hole in FBN-ARO-KO+GCI mice that received exogenous E2 replacement. Further quantitative analysis revealed that exogenous E2 replacement strongly decreased the primary escape latency (*F* = 69.65, *p* < 0.001 for FBN-ARO-KO; *F* = 49.267, *p* < 0.001 for E2 treatment; *F* = 47.549, *p* < 0.001 for FBN-ARO-KO and E2 treatment interaction; *p* < 0.001, *n* = 8, two-way ANOVA) and exploring errors (*F* = 21.322, *p* < 0.001 for FBN-ARO-KO; *F* = 9.893, *p* = 0.004 for E2 treatment; *F* = 7.528, *p* = 0.01 for FBN-ARO-KO and E2 treatment interaction; *p* = 0.003, *n* = 8, two-way ANOVA), and increased the quadrant occupancy (*F* = 25.006, *p* < 0.001 for FBN-ARO-KO; *F* = 17.322, *p* < 0.001 for E2 treatment; *F* = 9.1, *p* = 0.005 for FBN-ARO-KO and E2 treatment interaction; *p* = 0.002, *n* = 8, two-way ANOVA) in FBN-ARO-KO+GCI mice to levels that there were not significantly different from those observed in FLOX+GCI mice (Fig. 12*B*). These findings provide further evidence that reinstating brain E2 levels is able to rescue cognitive function in FBN-ARO-KO+GCI mice. Similar escape velocities among groups indicate that the observed differences were not because of speed variations. Hippocampal-dependent recognition memory was further investigated using the Novel Object Recognition Test (Fig. 12*C*). Similar exploring time on two identical objects in the sampling stage suggested that all the mice were not biased to the same objects that were located in different positions (*t* = −0.479, *p* = 0.639 for FLOX+GCI; *t* = −0.0853, *p* = 0.933 for KO+GCI; *t* = −0.267, *p* = 0.793 for KO+GCI+PL; *t* = −0.389, *p* = 0.703 for KO+GCI+E2; *n* = 8, unpaired *t* test; Fig. 12*Da*). However, in the choice stage, we observed a significantly reduced preference to the novel object in FBN-ARO-KO+GCI mice (*t* = −0.694, *p* = 0.499, *n* = 8, unpaired *t* test; Fig. 12*Db*) compared with FLOX+GCI mice (*t* = −4.179, *p* < 0.001, *n* = 8, unpaired *t* test; Fig. 12*Db*), indicating worse impairment in recognition memory. Conversely, E2 replacement strongly reversed the deficits, as evidenced by the increased exploring time on novel object (*t* = −3.324, *p* = 0.005, *n* = 8, unpaired *t* test; Fig. 12*Db*) and elevated discrimination index (*F* = 14.153, *p* < 0.001 for FBN-ARO-KO; *F* = 7.485, *p* = 0.011 for E2 treatment; *F* = 5.023, *p* = 0.033 for FBN-ARO-KO and E2 treatment interaction; *p* = 0.01, *n* = 8, two-way ANOVA; Fig. 12*Dc*).

## Discussion

While it has been known for some time that neurons make E2, the precise roles of neuron-derived E2 in the brain are not well understood. Previous work by our group demonstrated that neuron-derived E2 has a critical role in regulating forebrain synaptic plasticity and cognitive function in the male and female brain in the basal, noninjured state (Lu et al., 2019). In the current study, we used the same FBN-ARO-KO mouse model to examine the roles of neuron-derived E2 in the ischemic male and female brains. The results from our study suggest that neuron-derived E2 has several key roles following ischemic brain injury, including the following: (1) being critical for astrocyte activation in the hippocampal CA1 region; (2) suppressing neuronal FGF2 signaling as a mechanism to facilitate astrocyte activation; (3) enhancing expression of astrocyte-derived neuroprotective neurotrophins BDNF and IGF-1, and the glutamate transporter GLT-1; and (4) exerting neuroprotection and preserving hippocampus-dependent cognitive functions. It should be mentioned that loss of forebrain neuronal aromatase leads not only to E2 depletion in neurons but also to accumulation of androgen precursors. We believe the defects in FBN-ARO-KO mice are primarily because of loss of neuronal E2, as exogenous E2 replacement was able to fully rescue the defects in FBN-ARO-KO mice after GCI.

### Neuron-derived E2 is critical for astrocyte activation after ischemic injury

To our knowledge, this is the first report to demonstrate that neuron-derived E2 is critical for astrocyte activation following ischemic injury. A hallmark of reactive astrocytes after ischemia is that they transiently become hypertrophic and highly express the intermediate filaments, GFAP and vimentin (Sofroniew, 2009; Hamby and Sofroniew, 2010; Hol and Pekny, 2015). Mice deficient in GFAP and vimentin display attenuated astrocyte hypertrophy and reactive gliosis after brain injury (Li et al., 2008). In our study, FBN-ARO-KO mice displayed a significant decrease in astrocyte hypertrophy, as well as a robust decrease of GFAP and vimentin in the hippocampal CA1 region following GCI. Furthermore, RNA-sequencing analysis revealed significant alterations in RhoA signaling in FBN-ARO-KO mice, which has been shown to constrain actin motility and astrocyte reactivity, and is tightly controlled by STAT3, a factor whose expression and activation are known to be critical for induction of the reactive astrocyte phenotype (Chen et al., 2006; O'Callaghan et al., 2014; Renault-Mihara et al., 2017).

An interesting finding of our study is that elevation of astrocyte aromatase and hippocampal E2 levels after GCI was significantly attenuated in FBN-ARO-KO mice. We believe this effect is likely because of the decreased activation of astrocytes we observed in FBN-ARO-KO mice, as aromatase induction is known to occur only in activated astrocytes, and is not observed in basal, nonactivated astrocytes (Garcia-Segura et al., 1999; Q. G. Zhang et al., 2014). Further support of this suggestion comes from our finding that reinstating astrocyte activation in FBN-ARO-KO mice (by blocking FGF2 signaling) rescued the attenuated aromatase levels in hippocampal astrocytes after GCI.

### Role of neuronal FGF2 in neuron-derived E2-regulated astrocyte activation

The findings of our study further suggest that neuron-derived E2 facilitates astrocyte activation after GCI by restraining neuronal FGF2 signaling. RNA-Seq analysis showed increased FGF2 mRNA levels in FBN-ARO-KO mice after GCI, and double immunohistochemistry revealed that the FGF2 protein was significantly elevated in hippocampal neurons in FBN-ARO-KO mice after GCI. Neuron-derived FGF2 has been shown to exert an inhibitory effect on astrocyte activation by suppressing GFAP expression in astrocytes (Reilly et al., 1998; Y. Zhang et al., 2017). Blocking the FGF2 receptor in astrocytes by immunoneutralization or conditional deletion invokes reactive astrogliosis basally, and strongly enhances it after injury (Kang et al., 2014; Y. Zhang et al., 2017), indicating that FGF2 is an important signal to restrain induction of reactive astrocytes. FGF2 signaling appears to play an important role in the decreased astrocyte activation observed in FBN-ARO-KO mice in our study, as blocking FGF2 signaling by administration of a FGFR3-neutralizing antibody into the lateral ventricle of FBN-ARO-KO mice fully rescued astrocyte activation after GCI. In addition, aromatase, pSTAT3, BDNF, and GLT-1 expression was also rescued in astrocytes, followed by a significant reduction of neuronal damage after GCI. These findings strongly suggest that attenuated astrocyte activation in FBN-ARO-KO mice is due, at least in part, to enhanced neuronal FGF2 signaling.

### Neuron-derived E2 is neuroprotective

Our study also provides evidence that neuron-derived E2 exerts neuroprotection following ischemic injury. FBN-ARO-KO mice had exacerbated hippocampal neuronal damage after GCI, and worse hippocampal-dependent cognitive function compared with FLOX controls. Since FBN-ARO-KO mice exhibited attenuated reactive astrogliosis after GCI, it raises the possibility that the neuroprotective effect of neuron-derived E2 could be mediated by reactive astrocytes after GCI. Previous work suggests that reactive astrocytes can be either detrimental or beneficial. For instance, in cases of severe damage, astrocytes can become proliferative and form a scar, which could inhibit axonal regeneration after injury (H. Wang et al., 2018). However, our GCI model produces only mild injury and does not develop a scar. In contrast, a number of studies suggest that reactive astrocytes exert neuroprotection after ischemic injury (Li et al., 2008; Hayakawa et al., 2010). For instance, inhibition of reactive astrocytes with fluorocitrate retards neurovascular remodeling and recovery after focal cerebral ischemia (FCI) in mice (Hayakawa et al., 2010). In addition, GFAP^−/−^Vm^−/−^ mice exhibit attenuated reactive astrogliosis, increased neuronal damage, and reduced GLT-1-mediated glutamate transport after FCI (Li et al., 2008). This is highly similar to the phenotype of our FBN-ARO-KO mice after GCI, which have strong attenuation of GFAP and vimentin in the hippocampus, attenuated reactive astrogliosis, increased neuronal damage, and reduced hippocampal astrocytic GLT-1 levels.

Reactive astrocytes can protect neurons through several mechanisms, including increased uptake of excess glutamate and release of neuroprotective factors, such as BDNF, IGF-1, and even E2, itself (Verma et al., 2010; Arevalo et al., 2015; Z. Liu and Chopp, 2016; Y. Liu et al., 2017). With respect to glutamate transport, GLT-1 is a major astrocyte-specific Na^+^-dependent glutamate transporter that is responsible for 90% of glutamate uptake in the brain (Kim et al., 2011). Glutamate excitotoxicity is known to be a major mechanism of neuronal damage and death after ischemia, and upregulation of GLT-1 in astrocytes has been shown to protect hippocampal CA1 neurons from GCI (Ouyang et al., 2007). Thus, regulation of GLT-1 in astrocytes could be one mechanism whereby neuronal-derived E2 exerts neuroprotection after ischemia.

In addition, following GCI, FBN-ARO-KO mice also showed significant reductions in expression of two other astrocytic factors that have been implicated to be neuroprotective: BDNF and IGF-1. For example, astrocyte-specific overexpression of IGF-1 protected hippocampal neurons following brain injury (Madathil et al., 2013), and IGF-1 rescued CNS neurons following hypoxic-ischemic injury (Gluckman et al., 1992). Likewise, astrocyte-derived BDNF administration reduced infarct damage after FCI (Schäbitz et al., 2000), and conditional BDNF delivery from astrocytes has also been shown to rescue memory defects, spine density and synaptic function in a mouse model of AD (de Pins et al., 2019). Thus, in addition to regulation of GLT-1, enhanced astrocytic BDNF and IGF-1 production could also contribute to neuron-derived E2 neuroprotective effects after GCI. Finally, many studies have suggested that E2 produced by reactive astrocytes can itself exert neuroprotection (Garcia-Segura et al., 1999, 2003; Walters and Saldanha, 2008). Thus, the attenuated astrocyte activation and reduced aromatase/E2 induction in astrocytes after GCI could also contribute to the enhanced neuronal damage in FBN-ARO-KO mice.

Finally, we cannot exclude the possibility of a direct protective effect of neuron-derived E2 on neurons, as E2 is known to be protective if added directly to neurons *in vitro* (Ishihara et al., 2016; Zhao et al., 2016). Nevertheless, we believe a role for reactive astrocytes in neuron-derived E2 neuroprotection is strongly supported based on the findings that (1) aromatase and E2 go up in reactive astrocytes after GCI (Q. G. Zhang et al., 2014), and that (2) reinstatement of astrocyte activation in FBN-ARO-KO mice after GCI (by blocking FGF2 signaling) rescued the attenuated aromatase levels in hippocampal astrocytes, as well as reinstated pSTAT3, BDNF, and GLT-1 levels in the astrocytes, and strongly reduced GCI-induced damage to hippocampal neurons. Furthermore, in other work, we showed that astrocyte-specific aromatase KO mice have the same phenotype after GCI as FBN-ARO-KO mice (e.g., attenuated astrocyte activation, increased neuronal damage, and worse cognitive outcome compared with FLOX mice), despite having normal neuronal aromatase expression (J. Wang et al., 2019). This suggests that neuron-derived E2 effects after GCI involve mediation by reactive astrocytes and astrocyte-derived E2.

In conclusion, our study demonstrated a critical role of forebrain neuron-derived E2 in neuroprotection against ischemic injury. We provide the first evidence that neuronal E2 is critical for induction of reactive astrocytes and their ability to produce astrocyte-derived neurotrophic factors, BDNF and IGF-1, and the glutamate transporter, GLT-1, after ischemic brain damage. The beneficial effects of neuronal-derived E2 in ischemic brain injury appear to be due, at least in part, to suppression of neuronal FGF2 signaling, which is a known suppressor of astrocyte activation. As a whole, the study significantly advances our understanding of the beneficial roles of neuron-derived E2 in the ischemic brain.
